# Photodissociation Dynamics in (N_2_)_*n*_^+^ Clusters

**DOI:** 10.1021/acs.jpca.5c05798

**Published:** 2025-09-25

**Authors:** John R. C. Blais, B. Wade Stratton, Nathan J. Dynak, Brandon M. Rittgers, D. J. Kellar, Michael A. Duncan

**Affiliations:** Department of Chemistry, University of Georgia, Athens, Georgia 30602, United States

## Abstract

(N_2_)_*n*_^+^ cluster ions are produced and cooled in a pulsed-discharge supersonic expansion and studied with UV laser photodissociation and velocity-map imaging (VMI). All cluster sizes up to *n* = 15 absorb strongly near 355 nm, and those with *n* > 3 dissociate to produce both N_2_^+^ and N_4_^+^ photofragments. This suggests that the N_4_^+^ ion is the chromophore in the larger clusters, consistent with the previous optical spectroscopy and bond energy determinations. Photofragment imaging of N_4_^+^ produces an anisotropic distribution peaked along the laser polarization. Analysis of the maximum kinetic energy release produces a dissociation energy consistent with values determined in previous experiments. Dissociation of larger clusters produces N_2_^+^ with significant kinetic energy values that do not change appreciably with cluster size. This suggests that the N_4_^+^ core ion is not enclosed by the clustering of additional N_2_ molecules. N_4_^+^ fragments from larger clusters have somewhat lower kinetic energies than the N_2_^+^ fragments, consistent with recombination or partial caging after dissociative recoil. However, the kinetic energy release of N_4_^+^ is also considerable and it persists in the dissociation of larger clusters. This suggests that the N_4_^+^ ion in these clusters resides near the surface and that the photodissociation and recombination are mediated by this surface rather than by a true caging effect.

## Graphical abstract

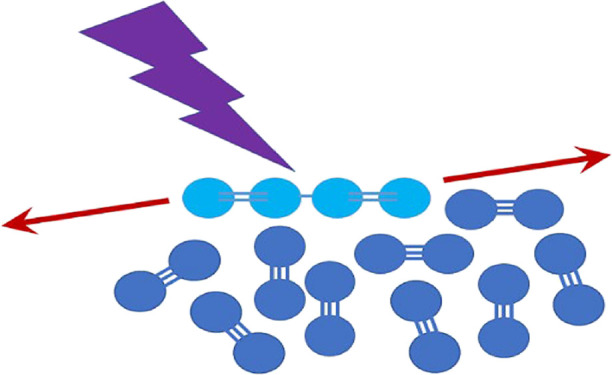

## Introduction

Nitrogen positive ions and their clusters play significant roles in terrestrial and planetary atmospheres,^1–3^ and these species have been well-studied in mass spectrometry and gas phase ion chemistry.^4–10^ Nitrogen cations and their clusters have also been investigated with infrared and optical spectroscopy, as well as computational chemistry.^11–27^ Like several other small atomic and molecular species (e.g., argon, O_2_, CO, CO_2_), ionization of N_2_ clusters produces a dimer positive ion with partial covalent character. Similar to the behavior of other covalent dimers, the N_4_^+^ cation is believed to survive as the core ion in larger clusters and to be the chromophore for their photoabsorption.^17,18,21,22^ Therefore, the larger ions are more properly indicated as N_4_^+^(N_2_)_*n*_ complexes. The spectroscopy and photodissociation behavior of small nitrogen cluster ions have been studied previously, documenting resonance wavelengths, fragmentation channels and bonding energetics.^4,7,9,11–27^ In the present work we use ion photofragment imaging to investigate the dynamics of photodissociation in these systems.

The electronic structure, bonding and spectroscopy of the N_4_^+^ ion have been investigated extensively.^4–27^ The bond energy has been determined by collision-induced dissociation, equilibrium experiments, photoion-photoelectron coincidence, and photofragment kinetic energy release measurements.^4,7–9,13,16,21^ The consensus of these experiments is that the bond energy is about 1.1 eV (25.4 kcal/mol). High resolution infrared spectroscopy experiments^24,25^ and electron spin resonance^15^ measurements establish that the structure is linear with a ^2^Σ_u_^+^ electronic ground state. UV–visible photodissociation spectroscopy finds a broad resonance beginning at around 550 nm and extending upward in energy, peaking at about 330 nm.^13,17,18,21,22^ This has been assigned to a ^2^Σ_u_^+^ → ^2^Σ_g_^–^ transition with a repulsive upper state. This same resonance at 330 nm is observed for the N_6_^+^, N_20_^+^ and N_40_^+^ cations, suggesting that N_4_^+^ is the chromophore and that this core ion is solvated in larger clusters.^17,18,22^

Although there are many studies of the photochemistry and spectroscopy of small atmospheric ions, there are fewer investigations of the structures and dynamics of large clusters.^28–40^ Most of the available information comes from mass spectrometry, and far less from spectroscopy. CO_2_^–^(CO_2_)_*n*_ clusters have been studied with anion photoelectron spectroscopy, revealing an intracluster core ion structure transition in larger cluster sizes.^29^ Protonated water clusters have been investigated with infrared photodissociation spectroscopy, and there are extensive studies of their structures and dynamics.^30^ Another interesting example includes ion mobility measurements of acetylene cluster ions, which suggested an intracluster reaction to form benzene.^32^ Likewise, anion water clusters have been examined to determine the location of the excess electron.^33^ Several examples have been found for intracluster reactions in larger molecular clusters containing metal ions.^31,34–36^ In some of the earliest work on cluster dynamics, Lineberger and co-workers conducted a series of studies on diatomic halogen anions clustered with CO_2_.^37–39^ These halogen anions dissociate on a repulsive excited state, with kinetic energy release into the fragment halogen atoms. These experiments found clear evidence for caging and recombination processes like those studied previously for the neutral halogens in solution.^41–45^ In a compelling size dependence behavior, smaller clusters allowed I and I^–^ atoms to escape after photodissociation of I_2_^–^, but larger clusters caged these atoms and they recombined to form I_2_^–^ after a time delay. Heaven and co-workers studied a similar system in the form of neutral I_2_Ar_*n*_ complexes.^40^ The present (N_2_)_*n*_^+^ system has some features in common with these halogen systems. The excited state of the N_4_^+^ core ion is repulsive, producing significant kinetic energy release and rotational excitation upon photodissociation.^13,21,23^ Bieske has studied the photodissociation products in larger cluster ions, which were found to consist of both N_2_^+^ and N_4_^+^ for sizes up to (N_2_)_7_^+^.^22^ In the present work, photofragment imaging is employed for the first time to investigate the dissociation and recombination dynamics in this system and how it depends on the size of the cluster ions.

## Methods

(N_2_)_*n*_^+^ ions are produced by a pulsed high voltage discharge in a supersonic nozzle expansion of pure nitrogen.^46^ Needle electrodes with a 0.5 mm gap are situated about 5 mm downstream of the nozzle aperture and pulsed at a level of 600–700 V (DEI model PVX-4140 pulser) for about 30 μs in the center of a 250 μs gas pulse of pure nitrogen. Previous work on other ions produced in this way found that optimized cooling is achieved when the high voltage is pulsed, as described here, rather than applied throughout the duration of the gas pulse. Ions produced in this way are typically believed to have rotational temperatures of 10–50 K. In some experiments, a small amount of water vapor was added to the expansion gas to improve ionization and cluster yields.^46^ The ions were analyzed and mass selected for study with a reflectron time-of-flight mass spectrometer designed for photodissociation experiments.^47,48^ Mass selection is accomplished with pulsed deflection plates in the first flight tube of the reflectron instrument, photodissociation takes place at the turning point in the reflectron field, and fragment mass analysis is accomplished using the flight time through a second drift-tube section. The photodissociation laser is a Nd:YAG (Continuum SureLite EX) operating on the third harmonic wavelength (355 nm) at a pulse energy of about 1–10 mJ/pulse in a spot size about 5 mm diameter.

Photofragment imaging studies were conducted using our selected-ion velocity-map imaging (SI-VMI) instrument.^49^ This instrument follows the many developments in the study of neutral photodissociation dynamics with imaging,^50–57^ and applies the same concepts to the study of jet-cooled ions. Our imaging instrument is a modified version of the reflectron time-of-flight spectrometer described above. In this configuration, ions are selected by their flight time though a linear time-of-flight section and then transmitted through the grounded reflectron assembly into an in-line imaging flight tube where they are decelerated and photodissociation occurs. The photodissociation laser is the same Nd:YAG mentioned above using pulse energies of 1–3 mJ/pulse. Photofragment ions are reaccelerated using a series of electrostatic lenses designed for velocity map imaging (VMI).^55^ The images of N_4_^+^ photodissociation were collected using the DC-slice imaging method.^56^ To achieve slicing, the dual MCP/P-47 phosphor detector (Beam Imaging Solutions BOS-75) is activated in a narrow time window with a fast rise-time high voltage pulser (DEI PVX-4140), allowing fragment ions in the central ∼90 ns of the arrival-time distribution to be detected. For the larger clusters, selected images were recorded with and without slicing, finding no significant differences. The images presented here are those without slicing. Images are collected using a CCD camera (Edmund Optics), averaging over several hundred thousand laser shots. Images are processed with the NuACQ and BasisFit software.^57^ Calibration was accomplished by measuring the image of Ar^+^ from the photodissociation of Ar_2_^+^ using the same instrument settings.^58^ The design for this instrument using photofragment imaging of jet-cooled ions that are mass-selected is unique to our lab,^49,58–64^ but similar instruments have recently been reported by other groups.^65–68^

Computational studies on the nitrogen cluster ions were carried out with the Gaussian16 program package,^69^ using either MP2 or density functional theory (DFT) with the B3LYP functional. All calculations used the aug-cc-pVTZ basis set.^70^ All energetics, i.e., dissociation energies, were zero-point corrected using harmonic frequencies, and all structures were confirmed to be minima (or not) via examination of the vibrational frequencies.

## Results and Discussion

A typical mass spectrum of the nitrogen ions produced from the discharge source is presented in Figure 1, showing that cluster ions out to about (N_2_)_20_^+^ are produced. The mass spectrum varies in an understandable way with the gas pulse backing pressure, gas pulse duration, and the discharge voltage. The ions at masses between the (N_2_)_*n*_^+^ species are mixed clusters containing water and there is a small amount of odd-numbered nitrogen clusters. Figure 2 shows the photodissociation mass spectra when the (N_2_)_*n*_^+^ ions are mass selected and photodissociated at 355 nm. The photodissociation is presented as a difference spectrum, in which the intensity of the ion without laser excitation is subtracted from that with laser excitation. The depletion of the parent ion is shown as a negative peak and the photofragments are presented as positive peaks. As shown, all cluster sizes up to (N_2_)_15_^+^ produce both the N_2_^+^ and N_4_^+^ fragment ions. 355 nm was chosen as the excitation wavelength for these studies because of previous work showing that nitrogen cluster ions absorb and dissociate at this wavelength.^13,17,18,21,22^ A broad resonance for N_4_^+^ was documented centered at 330 nm, and N_6_^+^, (N_2_)_15_^+^ and (N_2_)_20_^+^ were shown to have similar resonances.^17,18^ Bieske used selected wavelengths in this region and found photodissociation for ions up to (N_2_)_14_^+^.^22^ These ions have therefore been concluded to be N_4_^+^(N_2_)_*n*_ species, i.e., nitrogen-solvated N_4_^+^ ions. As shown here, all of the clusters in our experiment also absorb strongly and fragment at 355 nm, confirming the resonance in this wavelength region. Other significant observations from previous work are the bond energies determined for the larger N_2_^+^(N_2_)_*n*_ clusters by Hiraoka and Nakajima.^7^ In their work, N_4_^+^ was found to have a bond energy of 25.8 kcal/mol, consistent with several values from previous work. However, the bond energies for the loss of N_2_ from larger clusters (*n* = 2–11) were much smaller in the range of 2.8–1.7 kcal/mol, gradually decreasing for the larger species. There was no evidence for any solvation shell closing in these data. Our photodissociation results and fragmentation products are completely consistent with this previous work,^7,17,18,22^ and therefore it is reasonable to conclude, as previous researchers did, that these clusters contain the N_4_^+^ core ion solvated by nitrogen molecules.

**Figure 1:**
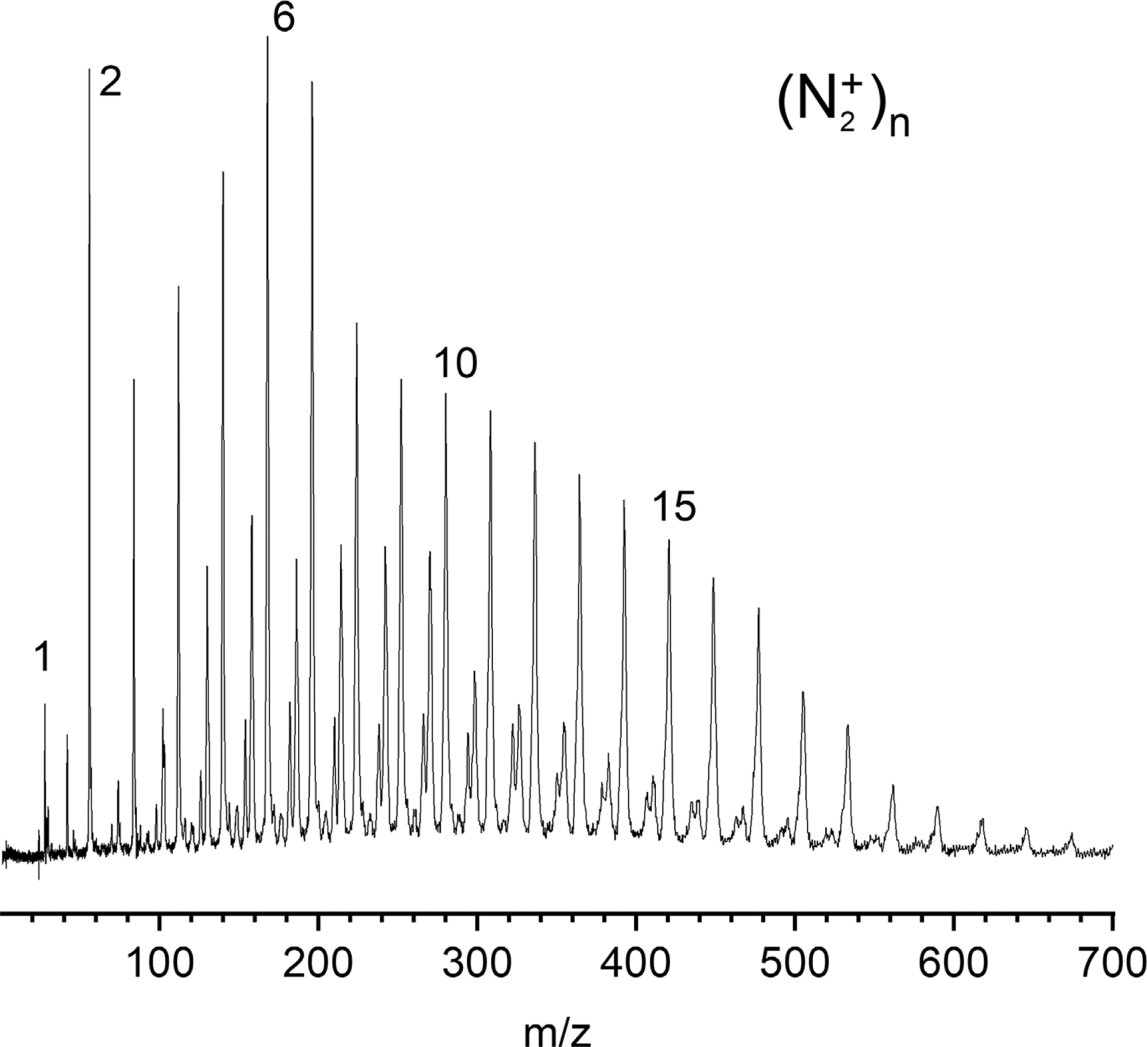
Mass spectrum of (N_2_)_*n*_^+^ ions produced by the pulsed-discharge supersonic nozzle source.

**Figure 2:**
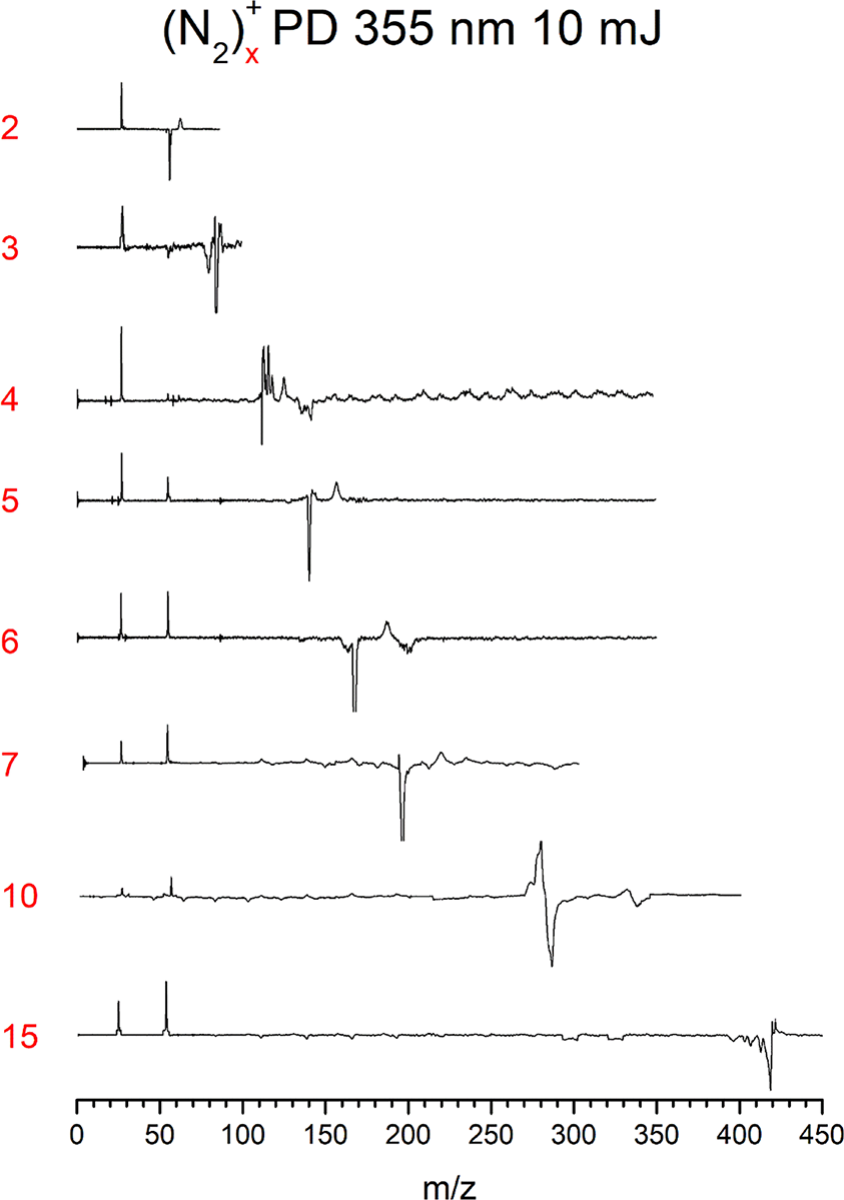
Photodissociation mass spectra of different-sized nitrogen cluster cations measured in the imaging instrument configuration. The negative-going peak indicates the depletion of the selected parent ion and the positive peaks indicate the photofragments coming from it. All cluster sizes except N_4_^+^ and N_6_^+^ produce both N_2_^+^ and N_4_^+^ as fragment ions. Because of mass discrimination effects and unstable cluster source, the integrated areas of the parent depletion and fragments are not equal.

The photodissociation products of these clusters are somewhat surprising. As seen previously by Bieske, the only fragment ions detected are N_2_^+^ and N_4_^+^.^22^ Bieske studied this in (N_2_)_*n*_^+^ cluster sizes up to *n* = 7, where he found that the relative yield of the N_4_^+^ fragment increased with increasing photon energy and with cluster size. We find the same trend with cluster size, although our quantitative branching ratios are somewhat different from those of Bieske, probably due to different mass spectrometer focusing and the variable collection efficiency for the fragment ions with significant kinetic energy release. In our reflectron instrument, when the fragment ions are formed in the turning region of the reflectron field, the N_2_^+^ and N_4_^+^ ions are both detected as fragments when smaller ions are dissociated. However, for the larger clusters we are unable to detect the N_2_^+^ and see only the N_4_^+^ fragment (see Figure S1). The detected branching ratios change when we use the imaging instrument (as in Figure 2), and the parent is decelerated without turning and both it and the fragment ions follow the same linear path to the detector. Additionally, the detector in the reflectron configuration has a small aperture (1 × 1 cm), whereas the detector in the imaging instrument is much larger (7 cm dia.). The different detection efficiency is therefore understandable if the N_2_^+^ fragment ions have significant kinetic energy release, which we find in the imaging experiment. These ions get thrown out of the beam in the reflectron configuration and do not make it to the detector, but the imaging instrument detects them because of its much larger detector area.

The formation of N_2_^+^ is expected from the dissociation of the N_4_^+^ chromophore, based on previous work.^13,17,18,20–22^ The formation of N_4_^+^ after excitation and dissociation of the N_4_^+^ chromophore in larger clusters suggests that some fraction of the N_2_^+^ fragment ions collide with the other nitrogen molecules in the cluster, resulting in a caging/recombination process, as discussed by Bieske.^22^ Significantly, there are no fragment ions detected for (N_2_)_*n*_^+^ species with *n* > 2, even for the largest clusters studied. This indicates that the N_2_^+^ ions are not completely caged, as a significant number escape, and that those which do recombine to form N_4_^+^ escape the cluster without binding to additional N_2_ molecules. It seems that this is only possible if the N_4_^+^ chromophore is either located near the surface of the cluster, or that there is so much excess kinetic energy that the remaining cluster is completely destroyed by the impact of photodissociation. The imaging experiments provide further insight into these issues.

### Photofragment Imaging

We use photofragment imaging to further investigate the dynamics of the dissociation processes in these clusters. We use the same laser wavelength (355 nm) indicated above, and detect the fragment ions N_2_^+^ and N_4_^+^ from these clusters using our photofragment imaging instrument. For the N_4_^+^ and N_6_^+^ ions, we image only the N_2_^+^ fragment, while for all the other ions we image both the N_2_^+^ and N_4_^+^ fragments. These images are presented in Figures 3–5.

**Figure 3:**
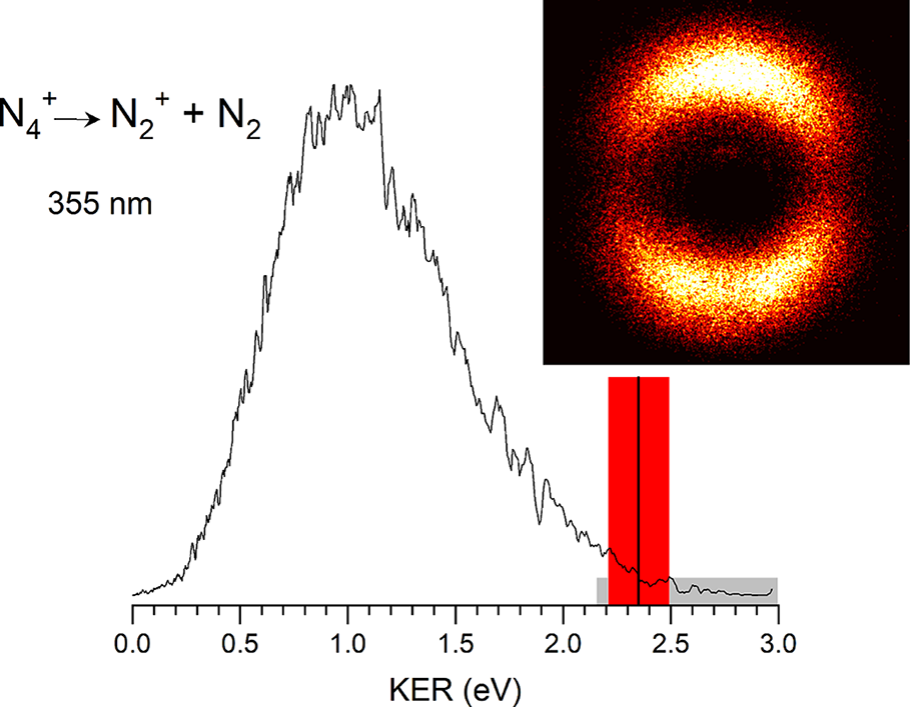
Photofragment image and fragment kinetic energy spectrum for the N_4_^+^ ion dissociating to produce N_2_^+^ (and N_2_), measured at 355 nm.

Figure 3 shows the image obtained for the N_2_^+^ fragment from the N_4_^+^ parent ion at 355 nm together with the analyzed kinetic energy spectrum. The kinetic energy spectrum represents the total kinetic energy in the two-fragment (N_2_^+^ + N_2_) system. An image for this same ion at 532 nm is presented in the Supporting Information as Figure S2. As shown, the image is strongly anisotropic with north–south intensity along the laser polarization. The angular distribution is well described with a β parameter of 1.42 ± 0.03 (see Figure S3). The image at 532 nm is somewhat less anisotropic, with a β parameter of 0.95 ± 0.02 (see Figures S4 and S5). Both images have a node at zero kinetic energy, indicating that essentially all ions have at least some kinetic energy release. However, both images are also broadened in the kinetic energy spectrum showing that there are considerable numbers of ions with somewhat less than the maximum kinetic energy, i.e., having internal energy from the dissociation process. These images make sense in terms of previous work on the photodissociation processes in this system. Jarrold et al. studied the kinetic energy release in a linear instrument configuration.^13^ Using photodissociation in the 458–514 nm region, they found β parameters in the 1.15–1.35 range. Bieske also studied the kinetic energy release in this system and found a similar broad spectrum for excitation at 488 nm.^21^

As shown in the kinetic energy spectrum in Figure 3, the N_2_^+^ kinetic energy ranges from about 0.3 to about 2.4 eV for photodissociation at 355 nm, with a maximum probability just above 1.0 eV. The bond energy for the dissociation of N_4_^+^ was determined in previous experiments to be about 1.1 eV (25.4 kcal/mol). Therefore, subtracting this and the most probable kinetic energy from the photon energy, most of the ions have about 3.49 eV −1.1–1.0 = 1.39 eV of internal energy. There is a broad distribution of kinetic energy corresponding to a broad distribution of internal energy, but the relative amounts of kinetic versus internal energy appear to be comparable. This internal energy production for the photodissociation of N_4_^+^ was investigated previously by Bieske using charge exchange reactions to detect vibrationally excited N_2_^+^, and also by Lessen et al. using laser-induced fluorescence spectroscopy on the N_2_^+^ photofragment^23^ which has a well-known electronic spectrum. Bieske found that about 30% of the N_2_^+^ fragments were produced in ν > 0 vibrational levels, making charge transfer to Ar possible.^21^ Lessen also found that a large amount of the N_2_^+^ ions were produced in the ground vibrational state (ν = 0) and that these ions had a significant amount of rotational excitation. Higher vibrational states were also detected, but the authors could not reconcile the total excess energy detected with the bond energy measured previously. Both Bieske and Lessen suggest that the previous measurements of the bond energy might have been affected by the internal energy of the N_4_^+^ ions. It is therefore interesting to consider what the present experiments, which employ jet-cooled ions, have to say about the bonding thermochemistry of N_4_^+^.

To investigate the bond energy of N_4_^+^, we consider what can be learned from the kinetic energy spectrum. With such a broad distribution of energy at all photodissociation wavelengths, it is conceivable, perhaps even likely, that some small fraction of the ions have essentially no internal energy and thus all the excess energy for these ions goes into kinetic energy release. Such ions would produce signal at the outside edge of the photofragment image. If ions like this are formed, and we have enough sensitivity to detect them, the maximum kinetic energy value (KER_max_) could be used in an energetic cycle to derive the bond energy. If all ions are produced with some internal energy, then the KER_max_ value would provide an upper limit on the bond energy. We therefore assign the KER_max_ value in Figure 3 using the red shaded box to represent the energy resolution of the instrument. This resolution was determined from the energy spectrum of Ar^+^ ions from the dissociation of Ar_2_^+^, where no internal energy is possible.^58^ We set the outside edge of this resolution element at the point where the signal rises above the background in the image, and then we set the determined KER_max_ value (indicated with the vertical black line) at the center of this resolution interval. The KER_max_ value for the dissociation at 355 nm is therefore 2.35 ± 0.14 eV. Assuming that this KER_max_ value represents ions with no internal energy, the photon energy (3.49 eV) minus this KER_max_ value (2.35 eV) gives the bond energy, i.e., *D*_0_ = 1.14 ± 0.14 eV (26.3 ± 3.6 kcal/mol). A similar analysis for our image at 532 nm produces a value of *D*_0_ = 1.13 ± 0.14 eV (26.1 ± 3.6 kcal/mol). These values compare to dissociation energies of 1.02 eV determined by Payzant and Kebarle,^4^ and 1.12 eV determined by Hiraoka and Nakajima,^7^ both obtained using equilibrium methods. Weitzel and Mähnert determined a value of 1.06 eV with a dissociative ionization method.^9^ Norwood et al. obtained 1.09 eV using photoion-photoelectron coincidence measurements.^16^ All of these previous values are within the error bars of the present measurements. In other recent studies of Fe^+^(acetylene) and Fe^+^(benzene) ion–molecule complexes, the KER_max_ values we determined at the maximum KER values of similar broad images corresponded to the actual bond energies determined in previous experiments using other methods.^61,62^ Because of this, and because we obtain essentially the same bond energy at two different wavelengths, we believe that our maximum KER values correspond to actual bond energies. Our bond energies are slightly higher, but consistent with those determined previously. This is understandable if the N_4_^+^ studied previously had some internal energy from the ion preparation, as suggested by Bieske^21^ and Lessen.^23^ For comparison to experiment, we performed computational studies on N_4_^+^ (details in the Supporting Information) at both the MP2 and DFT/B3LYP levels. The bond energy obtained at the MP2/aug-cc-pVTZ level is 1.295 eV (29.9 kcal/mol), although the linear structure that produces this number is not a stable minimum (see the Supporting Information). Computations at the DFT/B3LYP/aug-cc-pVTZ level find a stable linear structure whose dissociation energy is 1.748 eV (40.3 kcal/mol) (see the Supporting Information). Both of these values are higher than all of the experimental values. However, there are problems with the computations, as discussed further below. Handy and co-workers determined a bond energy of 1.21 eV (27.9 kcal/mol) using CCSD­(T) computations.^26^

Because the N_4_^+^ ion dissociates with such high kinetic energy release, it is interesting to consider what happens in larger clusters. We have therefore measured photofragment images for several larger cluster sizes, recording images for both the N_2_^+^ and N_4_^+^ fragment ions. These images are shown in Figures 4 and 5, along with their respective kinetic energy spectra. The angular distribution fits for these clusters are presented in the Supporting Information (Figures S2–S17). The determination of kinetic energy spectra for these larger clusters is not as straightforward as it is for the N_4_^+^ system because the detected ions are most likely recoiling from multiple neutral N_2_ photofragment partners. Because of the weak bonding between N_2_ units, there is no reason to expect that the neutral mass in these photofragment events is contained in a single multinitrogen cluster. Accounting for the total kinetic energy in the system therefore requires that the momentum and energy of these other fragments be accounted for. However, as shown in the Supporting Information, the assumption of a single neutral particle with the total mass of the multiple neutral N_2_ molecules moving as a unit in the center-of-mass frame provides a useful approximation for handling this issue, and that is how we have analyzed these systems. As explained in the SI, this analysis leads to a determination of the overall kinetic energy that is less than or equal to the actual value.

**Figure 4:**
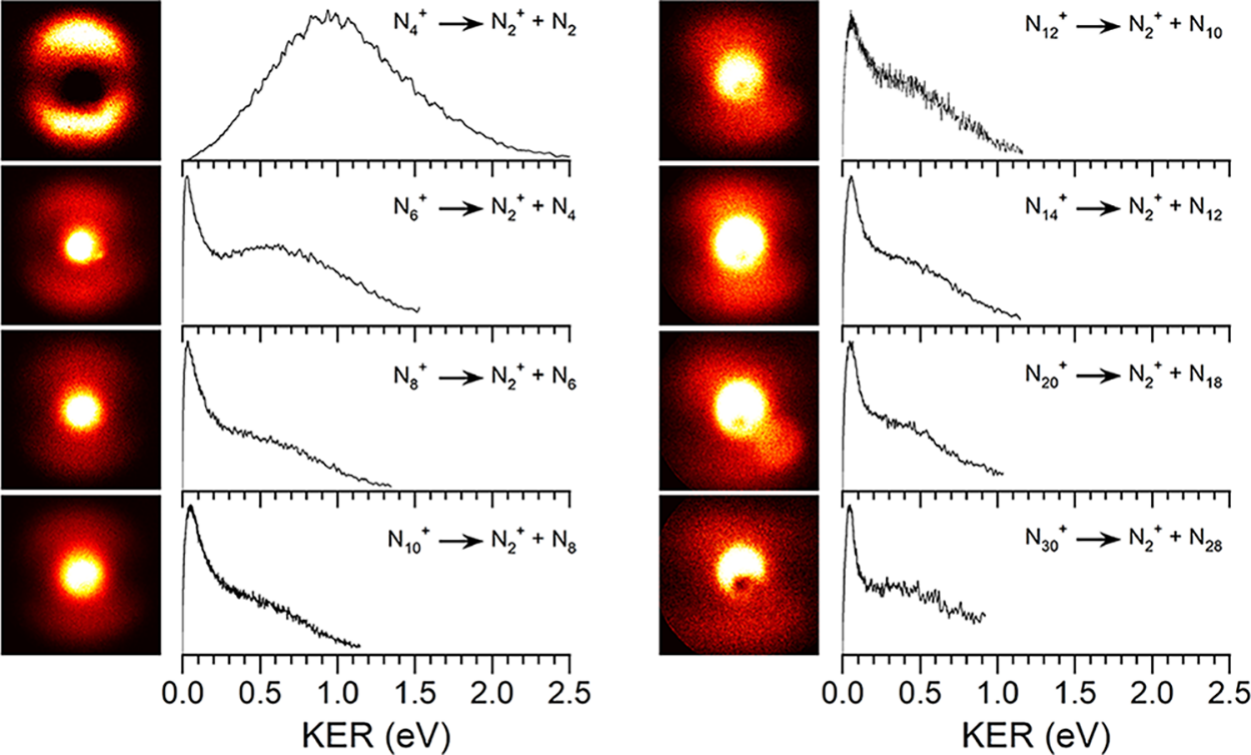
Photofragment images and fragment kinetic energy spectra for the (N_2_)_*n*_^+^ ions dissociating to produce N_2_^+^ and (*n* – 1)­(N_2_) neutrals, measured at 355 nm.

As shown in Figure 4, the N_2_^+^ fragment from each of the various (N_2_)_*n*_^+^ clusters is ejected with some kinetic energy. However, the resulting images are significantly different from that of the N_4_^+^ dissociation in that there is a bright spot at the center corresponding to zero or near-zero (0–0.2 eV) kinetic energy for each cluster size. Each image also has a higher energy signal in the 0.2–1.0 eV range, whose relative intensity declines gradually with cluster size. The higher energy signal is broad, indicating the presence of internal ro-vibrational excitation, but it is not as broad nor peaked at as high an energy value as the spectrum for the N_4_^+^ dissociation. Significantly, however, the higher energy signal persists throughout all the cluster sizes studied. The maximum kinetic energy observed is about 1.4 eV for N_6_^+^, about 1.2 eV for N_8_^+^, and then it is virtually unchanged at about 1.0 eV for all the larger clusters. The anisotropic angular distribution of the fragment N_2_^+^ ions is degraded in the fragmentation of larger ions, but persists to some degree in these systems.

Figure 5 shows the photofragment images and kinetic energy spectra of the N_4_^+^ ion produced in the dissociation of various (N_2_)_*n*_^+^ clusters. As shown in Figure 3, there is no significant production of N_4_^+^ as a fragment from N_6_^+^, and so the smallest parent ion studied in this way is N_8_^+^. Each of these images has a bright center spot corresponding to zero or near-zero kinetic energy, which is reflected in the kinetic energy spectra. The N_8_^+^ image has a wider spread in the low energy peak, whereas all larger clusters have most of their signal in the 0.0–0.4 eV range. While this low energy feature explains the majority of the signal for all these clusters, they all have a smaller component of higher energy signal in the 0.5–1.0 eV range. None of these N_4_^+^ images has any discernible anisotropy like that seen for the N_2_^+^ fragment ions in Figure 4.

**Figure 5:**
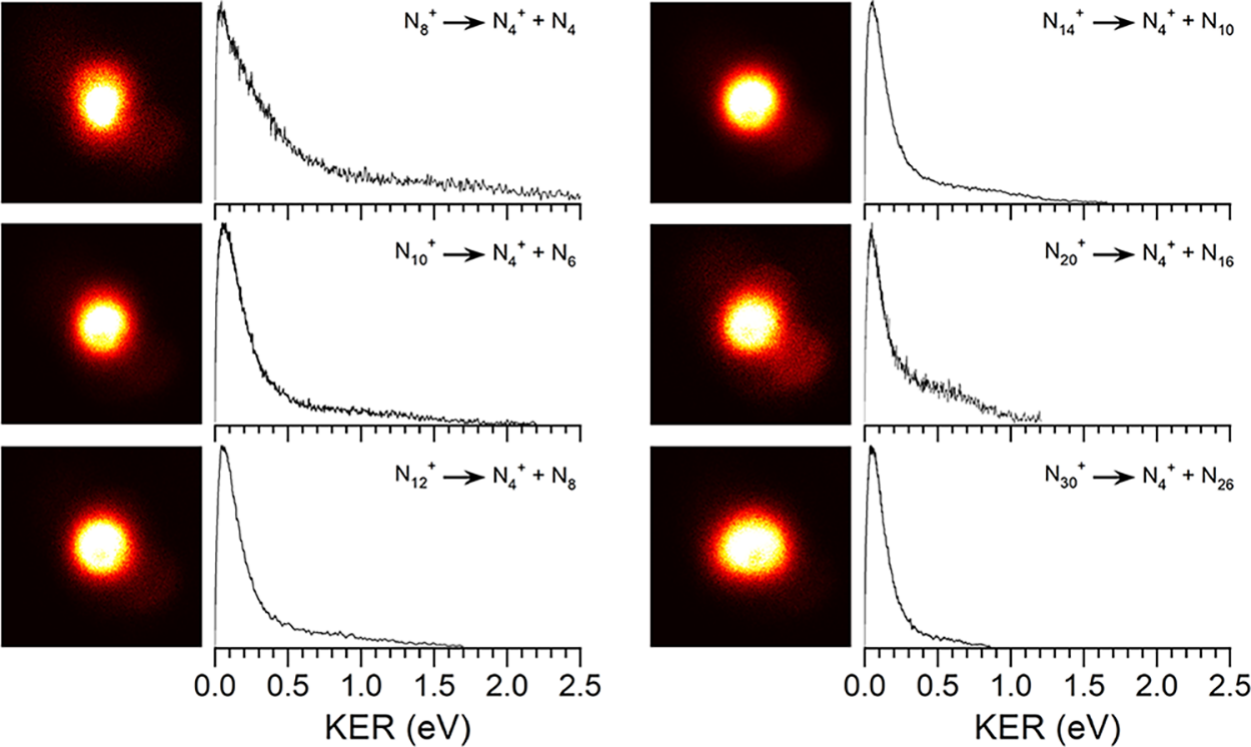
Photofragment images and fragment kinetic energy spectra for the (N_2_)_*n*_^+^ ions dissociating to produce N_4_^+^ and (*n* – 2)­(N_2_) neutrals, measured at 355 nm.

The anisotropy in these images varies with the kinetic energy of the fragments. This is most apparent for the images of the N_2_^+^ fragments, which each have a high energy component in their KER spectra. In Figure 6, we dissected the images of the N_2_^+^ fragments from the N_6_^+^ and N_14_^+^ ions into low energy and high energy components. These two component images were then fit to angular distributions. As shown in the figure, the low energy components have low anisotropy, consistent with collisional quenching. The high energy components have significant anisotropy, consistent with direct ejection from the cluster without significant collisional energy loss.

**Figure 6:**
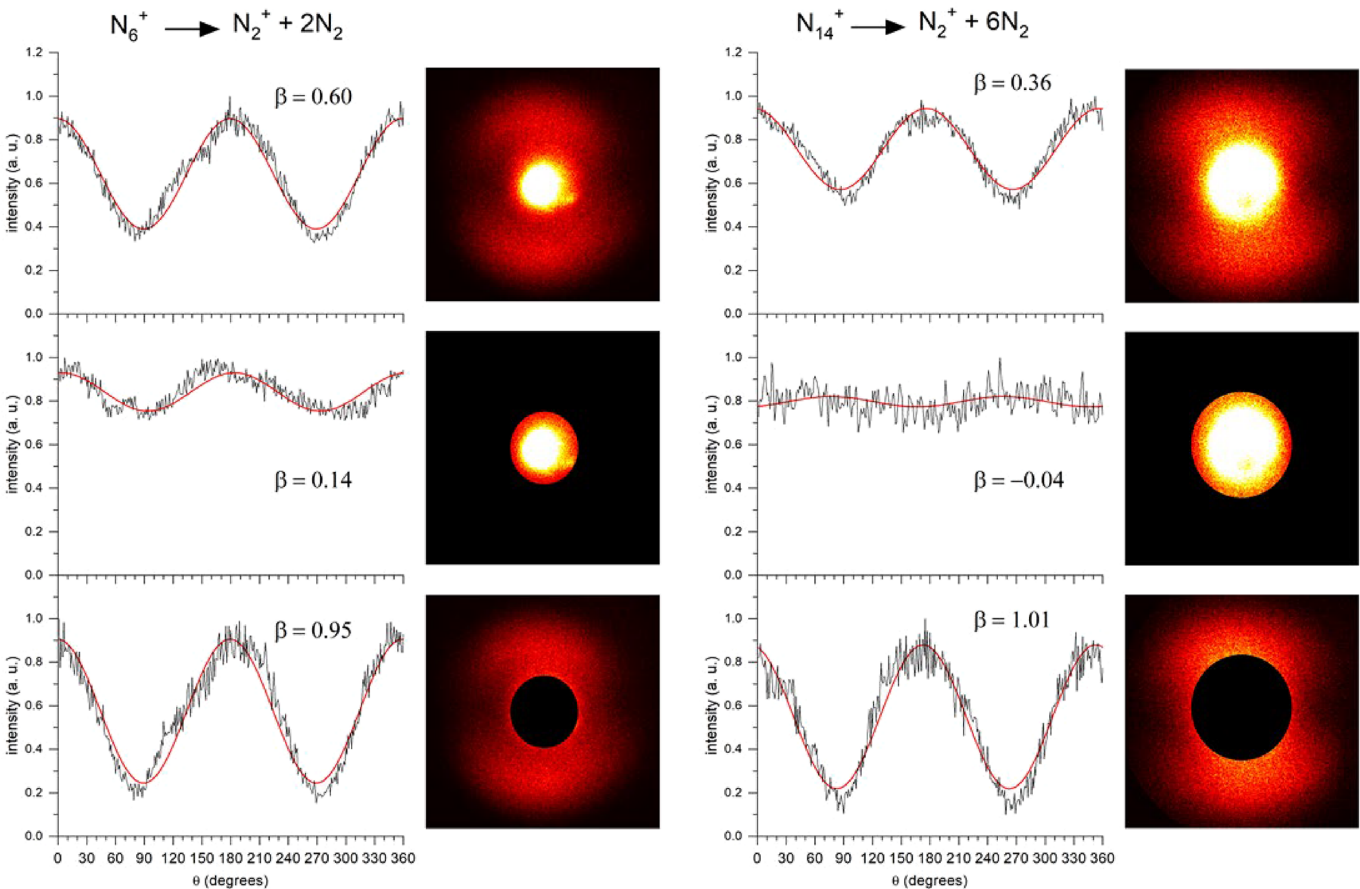
Photofragment images and angular distributions for N_6_^+^ and N_14_^+^ measured at 355 nm. The upper frame in each set shows the angular distribution when ions of all kinetic energy values are included in the fits. The middle frames show the angular distribution of the low KER fraction of the signal, and the lower frames show the angular distribution of the high KER fraction of the signal. In both cases, the higher KER signal is much more anisotropic.

These images and their size dependence provide interesting insights into the photodissociation dynamics of these clusters. It is expected that dissociation of the N_4_^+^ core ion would produce kinetic energy in its N_2_ and N_2_^+^ fragments because of the previous work on this ion, and the image shown in Figure 3 is consistent with this. However, it is not initially clear how this kinetic energy would be accommodated in the larger clusters. As shown in Figure 4, the N_2_^+^ fragment continues to have significant kinetic energy release for all cluster sizes studied, but it also has a strong propensity for a fraction with lower kinetic energy. The low kinetic energy feature appears for the first time in the spectrum of N_6_^+^, and represents about half of the signal for all cluster sizes. This suggests that the recoil from the N_4_^+^ dissociation is cushioned via collisions with other N_2_ molecules in the cluster, in an effect related to “caging”. The moderation of the strong anisotropy in the distribution for larger clusters is consistent with this. Another possibility is resonant charge transfer between the initial N_2_^+^ fragment and other N_2_ molecules in the cluster. This would also produce N_2_^+^ ions with lower average kinetic energy and more isotropic angular distributions. The formation of the N_4_^+^ fragment ion is also consistent with collisional energy transfer following dissociation. It is not clear how this ion could be formed except by a collision between an ejected N_2_^+^ and the other N_2_ molecules in the cluster. Bieske concluded that linear structures for N_6_^+^ and N_8_^+^, with extra N_2_ molecules binding at either end of the core N_4_^+^, would likely explain the recombination process to form N_4_^+^.^22^ Such collisions are apparently energetic enough to displace the other N_2_ molecules, since no larger N_*n*_^+^ fragment ions are detected, even in the largest clusters. Hiraoka and Nakajima determined the binding energies of the third, fourth and fifth N_2_ molecule in these (N_2_)_*n*_^+^ clusters to be 2.76, 2.71, and 2.52 kcal/mol (0.12, 0.12, 0.11 eV) which are significantly less than that for the N_4_^+^ ion (25.8 kcal/mol; 1.12 eV).^7^ This is consistent with the picture of an N_4_^+^ core ion solvated by other N_2_ molecules and also consistent with the extensive fragmentation of the larger clusters.

The low kinetic energy fraction for N_2_^+^ ions at all cluster sizes and the formation of the N_4_^+^ fragment can only occur through collisional energy transfer, but these dissociation events do not appear to be consistent with “caging” as it is usually understood. In the di-halogen anions studies of Lineberger and co-workers, larger CO_2_ clusters produced structures which enclosed the halogen chromophore, so that the kinetic energy following photodissociation was effectively quenched, allowing complete recombination to occur eventually in the largest clusters.^37–39^ The amount of caging increased with cluster sizes. Caging might be expected in the present system, as it is easy to picture the N_4_^+^ ion symmetrically solvated by surrounding N_2_ molecules. However, if there were caging in the present system, we would expect only low kinetic energy for the N_2_^+^ fragments at larger cluster sizes and eventually only the formation of N_4_^+^ as a fragment. We might also expect the formation of fragment ions larger than N_4_^+^. However, the yield of N_4_^+^ changes only slightly with cluster size and N_2_^+^ is detected as a significant fragment for all cluster sizes. Additionally, a substantial fraction of the N_2_^+^ ions continue to have significant kinetic energy and anisotropy for all cluster sizes, as if they had no strong collisional energy transfer. Conversely, the N_4_^+^ ions have significantly lower kinetic energy (although not zero) and no anisotropy, as if all suffered some sort of collisions. If N_4_^+^ formed from recombination of the original N_4_^+^ core ion, its branching fraction should increase with cluster size and its kinetic energy should decrease more with cluster size. These do not happen, and so it is more likely that N_4_^+^ forms by N_2_^+^ recoil followed by reaction with other N_2_ molecules as the cluster shatters.

This fragmentation behavior suggests an intriguing picture of the structures of the larger clusters. The dynamics seem to be inconsistent with an enclosed/symmetrically solvated N_4_^+^ ion, but rather one which is instead most often situated on the surface of the cluster or asymmetrically immersed in it. “Surface” N_4_^+^ ions could explain why roughly half of the N_2_^+^ fragments are ejected with significant kinetic energy in an anisotropic distribution, even for the larger clusters. These ions would experience little or no collisional relaxation. Other N_2_^+^ fragments are ejected with low kinetic energy, consistent with “grazing incident” collisions as they leave the cluster. Another fraction of the ions are ejected more directly into the cluster, where they are collisionally relaxed and recombine to form N_4_^+^. Some of these retain small amounts of kinetic energy, but this fraction is reduced gradually with cluster size. Cluster structures consistent with this picture have been suggested previously for the N_6_^+^ ion, which was suggested to have the extra N_2_ bound in an end-on linear configuration rather than a “T” shape.^22^ We confirmed this structure in our DFT computations. A surface-ion structure, rather than a solvated-ion configuration, would also help to explain why all the different sized clusters have essentially the same absorption spectra. Although surface ions are not usually expected in clusters such as these, other examples are known, particularly in the case of magnesium and aluminum cation complexes.^71,72^ In these metal ion complexes, polarization of the metal orbitals causes an asymmetric charge center and solvent/ligand molecules bind opposite this.

It would of course be interesting to confirm these suggestions about cluster structures with computational studies on these systems. However, our attempts to do this have been challenging. MP2 calculations on N_4_^+^ found a bent structure with two N_2_ moieties in a “Z” configuration. A linear species (corresponding to the experimentally known structure) was not a stable minimum and had multiple imaginary frequencies. These problems were amplified in the N_6_^+^ ion, which also did not have a stable linear structure. Unphysically high frequencies were found for all structures as well as high <*S* ^2^> values indicating spin contamination. These MP2 data are therefore unreliable. DFT is known to be less sensitive to spin contamination and multireference problems, and so we also did computations at the DFT/B3LYP level. A stable linear structure was found for N_4_^+^, consistent with experiment, and a similar linear structure was found for N_6_^+^. Reasonable vibrational frequencies were found for both ions. A linear structure for N_6_^+^ would be completely consistent with its photofragment image, which had both high and low kinetic energy components. N_2_^+^ could be ejected away from the molecular axis or into it, explaining both components. However, the bonding energetics found with DFT were not so satisfying. The bond energy for linear N_4_^+^ was predicted to be 40.3 kcal/mol and that for linear N_6_^+^ was 12.4 kcal/mol; both are much higher than the experimental values. Similar calculations for N_8_^+^ and N_10_^+^ found both linear and nonlinear structures (see Supporting Information), whose relative energies were quite small - likely less than the accuracy of the DFT calculations. Because of this, we did not pursue calculations on any larger ions. The incremental bond energies for N_*n*–2_^+^-N_2_ dissociation of linear species decrease gradually with cluster size. These linear structures could also be consistent with the imaging data, if we assume that N_4_^+^ is at the end of the linear structure. However, NBO analysis for these clusters show that charge is distributed evenly over the structure, i.e., this is *not* consistent with the integrity of an N_4_^+^ ion as suggested by the optical spectroscopy and the experimental bond energies. From these initial computational studies, we can conclude that reliable theory on this system may require more advanced methods than those employed so far. In the future, it may be possible to use infrared spectroscopy to further elucidate these cluster structures.

## Conclusions

Photofragment imaging has been employed to investigate the photodissociation dynamics of nitrogen cluster ions. Imaging of the photodissociation of jet-cooled N_4_^+^ at 355 and 532 nm is consistent with previous studies using other methods at other wavelengths, indicating a combination of kinetic energy release and internal excitation of the N_2_^+^ fragment. Strongly anisotropic images are obtained at both wavelengths, consistent with dissociation off a repulsive excited state potential, as has been suggested previously. An energetic cycle using the maximum kinetic energy obtained provides new estimates for the dissociation energy of this ion, which are slightly revised to higher energy compared to previous work. This difference likely results because the present ions are colder than those studied before.

Larger nitrogen cluster ions dissociate via a common resonance independent of cluster size, also consistent with previous work which has assigned these ions to have the N_4_^+^ core ion as the chromophore. N_2_^+^ and N_4_^+^ fragment ions are detected for all cluster sizes, extending the previous work. In a new approach, photofragment imaging is also applied to these systems, with surprising results. The N_2_^+^ fragment ions have both high and a low kinetic energy components in their distributions for all cluster sizes. The anisotropy seen for N_2_^+^ fragments from N_4_^+^ photodissociation persists to some degree in all the larger clusters, and is prominent in the high energy component of the N_2_^+^ kinetic energy spectra. The N_4_^+^ photofragments have distributions with mostly low kinetic energy and a smaller component of high kinetic energy that do not change appreciably with cluster size. These observations suggest that the N_4_^+^ core ion resides at or near the surface of these clusters rather than being enclosed by solvation from the additional N_2_ molecules. Computational studies on large nitrogen cluster ions such as these are extremely challenging because of the many isomeric structures expected. Calculations may be able to elucidate the structural suggestions made here, but they would need to be done with methods more sophisticated than DFT.

Ion enrichment at aqueous interfaces has been explored in the context of atmospheric aerosols,^73^ and partial solvation of reactant ions near the surfaces of charged microdroplets has been implicated as a factor in their enhanced reactivity.^74,75^ Although these clusters are much smaller by comparison, the surface-solvated structures suggested here are also intriguing.

## Supplementary Material



## Notes

The full citation for ref 68 and the details of the computations done, including the structures and energetics for each of the cluster ions considered. Image for N_4_^+^ at 532 nm; angular distribution fits; kinetic energy analysis for multi-nitrogen cluster dissociation; and MP2 and DFT computational studies (PDF)
