# Supplementary material for: Photodissociation Dynamics in (N2) n + Clusters
Source: J Phys Chem A. 2025 Sep 25;129(40):9387–96. doi: 10.1021/acs.jpca.5c05798 (PMC12516711; doi:10.1021/acs.jpca.5c05798)
Supplement: Supplementary file 1 [file jp5c05798_si_001.pdf]

J. Phys. Chem. A

Supporting Information

Photodissociation Dynamics in $(N_2)_n^+$ Clusters

John R. C. Blais,[1] B. Wade Stratton,[1] Nathan J. Dynak,[1] Brandon M. Rittgers,[1] D. J. Kellar,[1] Michael A. Duncan*[1]

[1]Department of Chemistry, University of Georgia, Athens, Georgia 30602, United States

*Email: maduncan@uga.edu

Table of Contents

	Page
Image for N_4^+ at 532 nm	S2
Angular Distribution Fits	S3−S18
Kinetic Energy Analysis for Multi-Nitrogen Cluster Dissociation	S19
Computational Studies	S22
MP2 Computations	S23
DFT Computations	S27

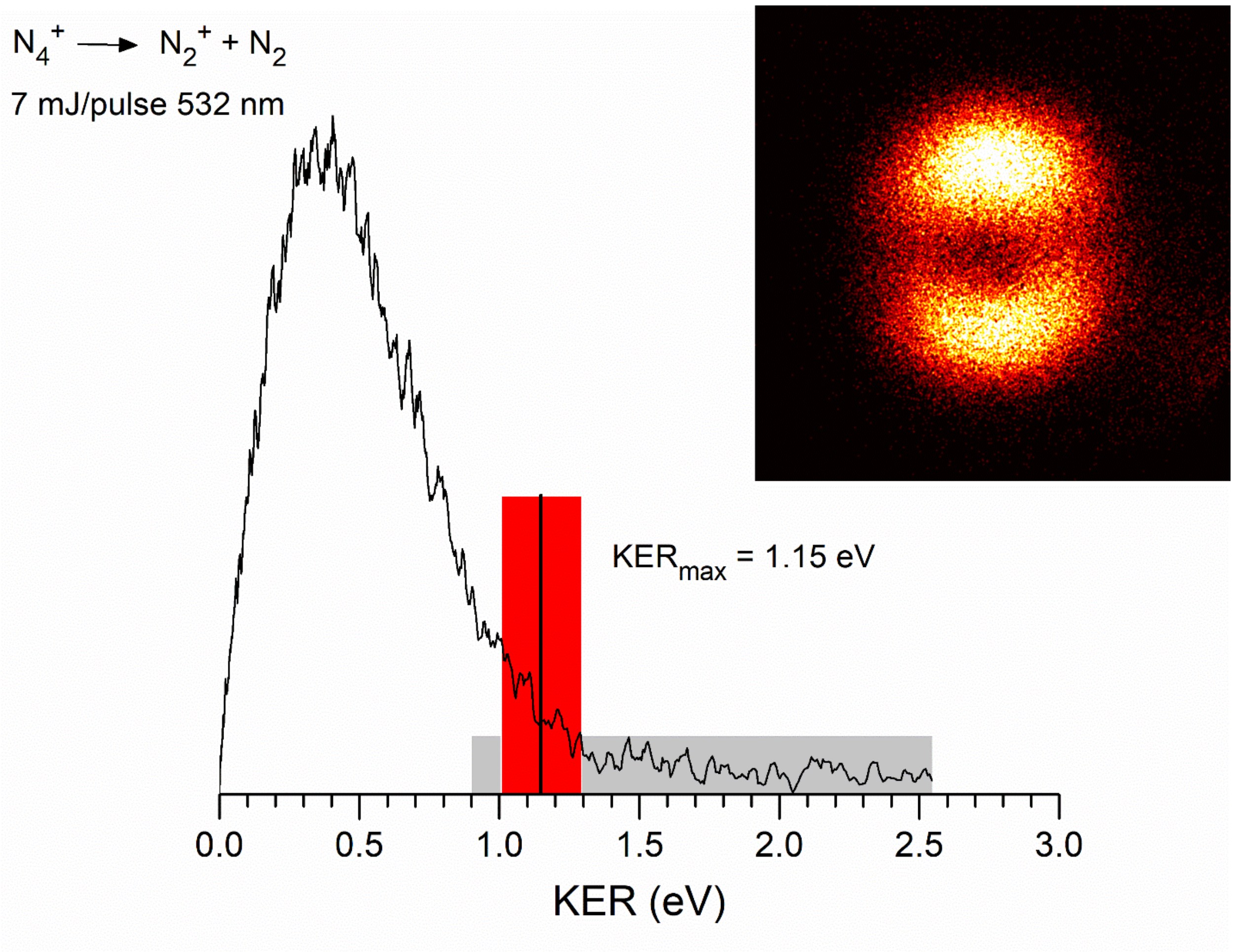

Figure S1. The total energy distribution of the sliced N_2^+ photofragment image from the dissociation of N_4^+ with vertically polarized light at 532 nm. The black vertical line indicates the assigned values for the maximum kinetic energy release, the red box indicates the width of the instrument resolution, and the gray box indicates the background signal.

Photofragment Angular Distributions

The following equation is used for fitting angular distributions:

$$I = \frac{A}{4\pi}\left\{1 + \frac{B}{2}\left[3\,cos^2\left(\frac{\theta\pi}{180} - C\right) - 1\right]\right\}$$

I is the signal intensity. The *A* parameter allows for variation of the amplitude, *B* is the β parameter, and *C* is a phase shift parameter correcting for rotation of the image.

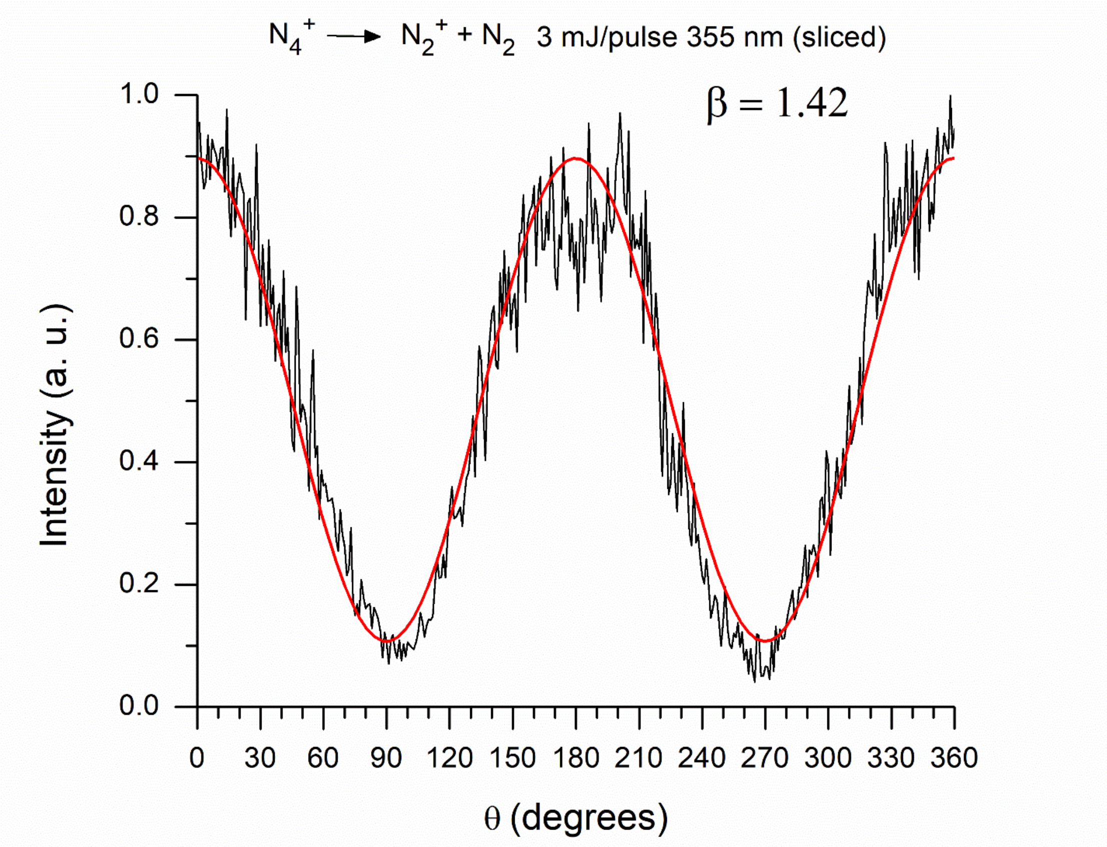

Figure S2. The angular distribution of the sliced N_2^+ photofragment image from the dissociation of N_4^+ with vertically polarized light at 355 nm. The red line is a fit with ß = 1.42.

A = 4.65384 $\pm$ 0.05564

B = 1.42216 $\pm$ 0.03164

C = $-$0.01700 $\pm$ 0.00717

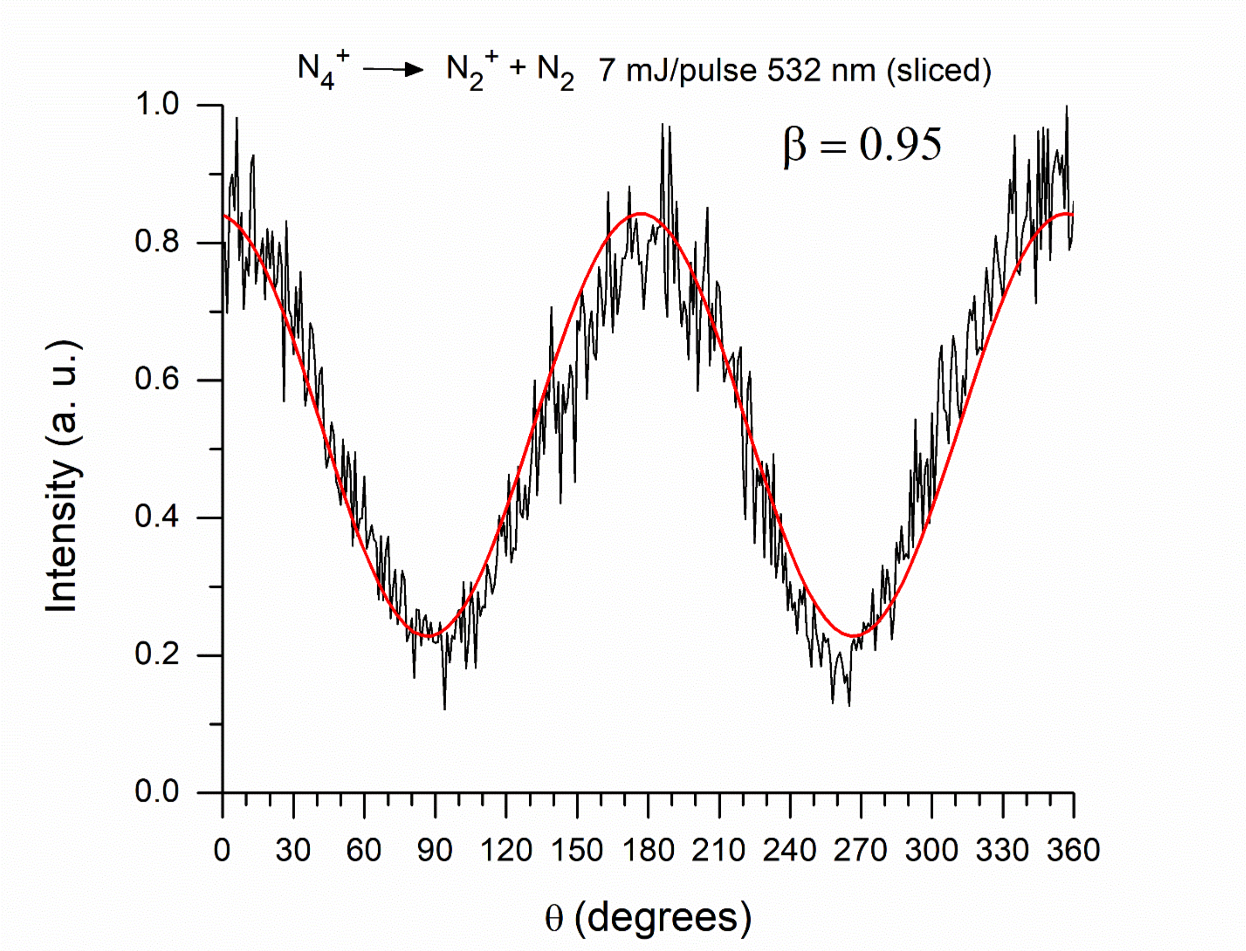

Figure S3. The angular distribution of the sliced N_2^+ photofragment image from the dissociation of N_4^+ with vertically polarized light at 532 nm. The red line is a fit with ß = 0.95.

A = 5.43852 ± 0.05452

B = 0.94714 ± 0.02283

C = − 0.05757 ± 0.00903

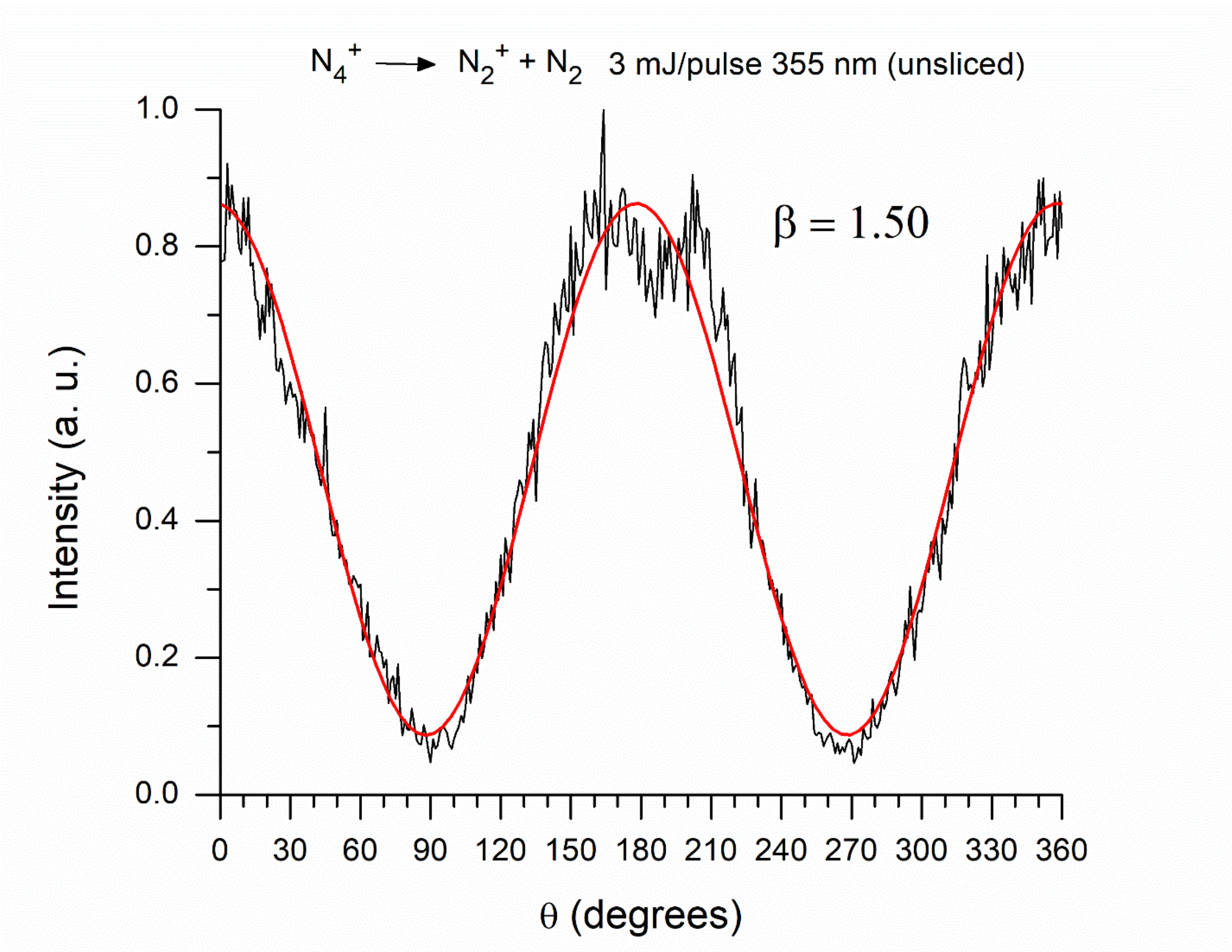

Figure S4. The angular distribution of the unsliced N_2^+ photofragment image from the dissociation of N_4^+ with vertically polarized light at 355 nm. The red line is a fit with ß = 1.50.

A = 4.34411 ± 0.03958

B = 1.49690 ± 0.02466

C = − 0.03462 ± 0.00519

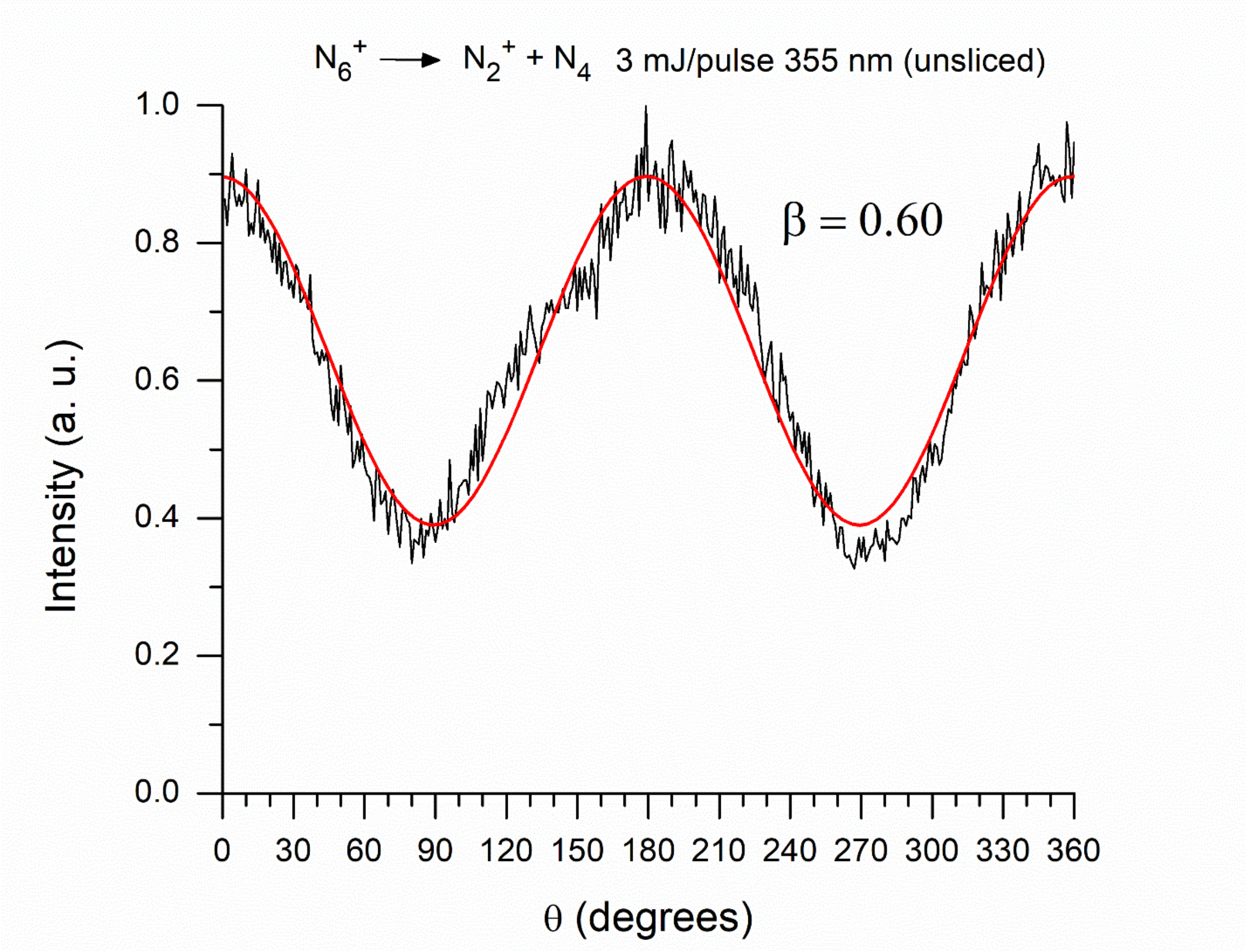

Figure S5. The angular distribution of the unsliced N_2^+ photofragment image from the dissociation of N_6^+ with vertically polarized light at 355 nm. The red line is a fit with ß = 0.60.

$A = 7.02316 \pm 0.03374$

$B = 0.60498 \pm 0.00979$

$C = -0.01443 \pm 0.00677$

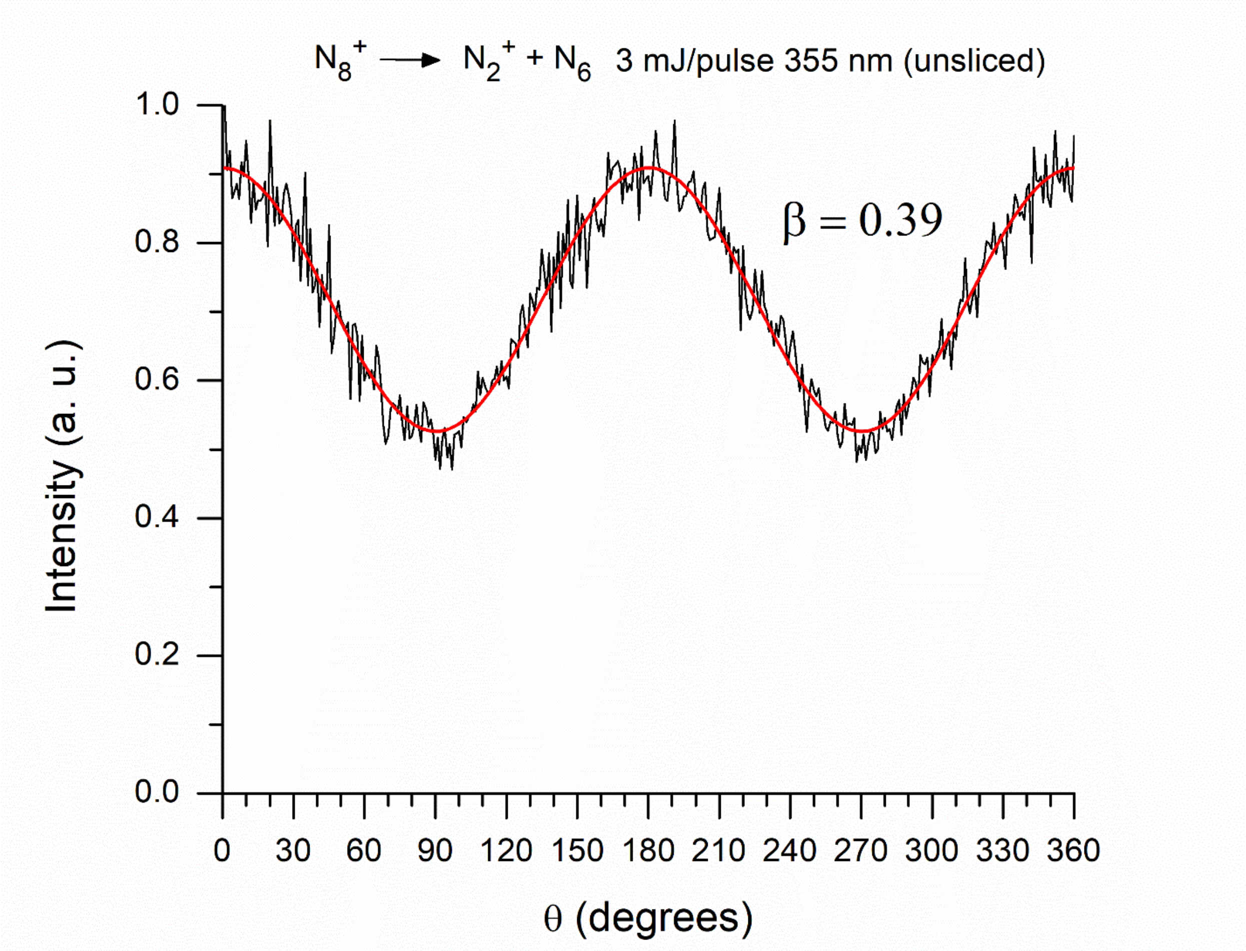

Figure S6. The angular distribution of the unsliced N_2^+ photofragment image from the dissociation of N_8^+ with vertically polarized light at 355 nm. The red line is a fit with ß = 0.39.

A = 8.21629 ± 0.02440

B = 0.39090 ± 0.00566

C = 0.00326 ± 0.00648

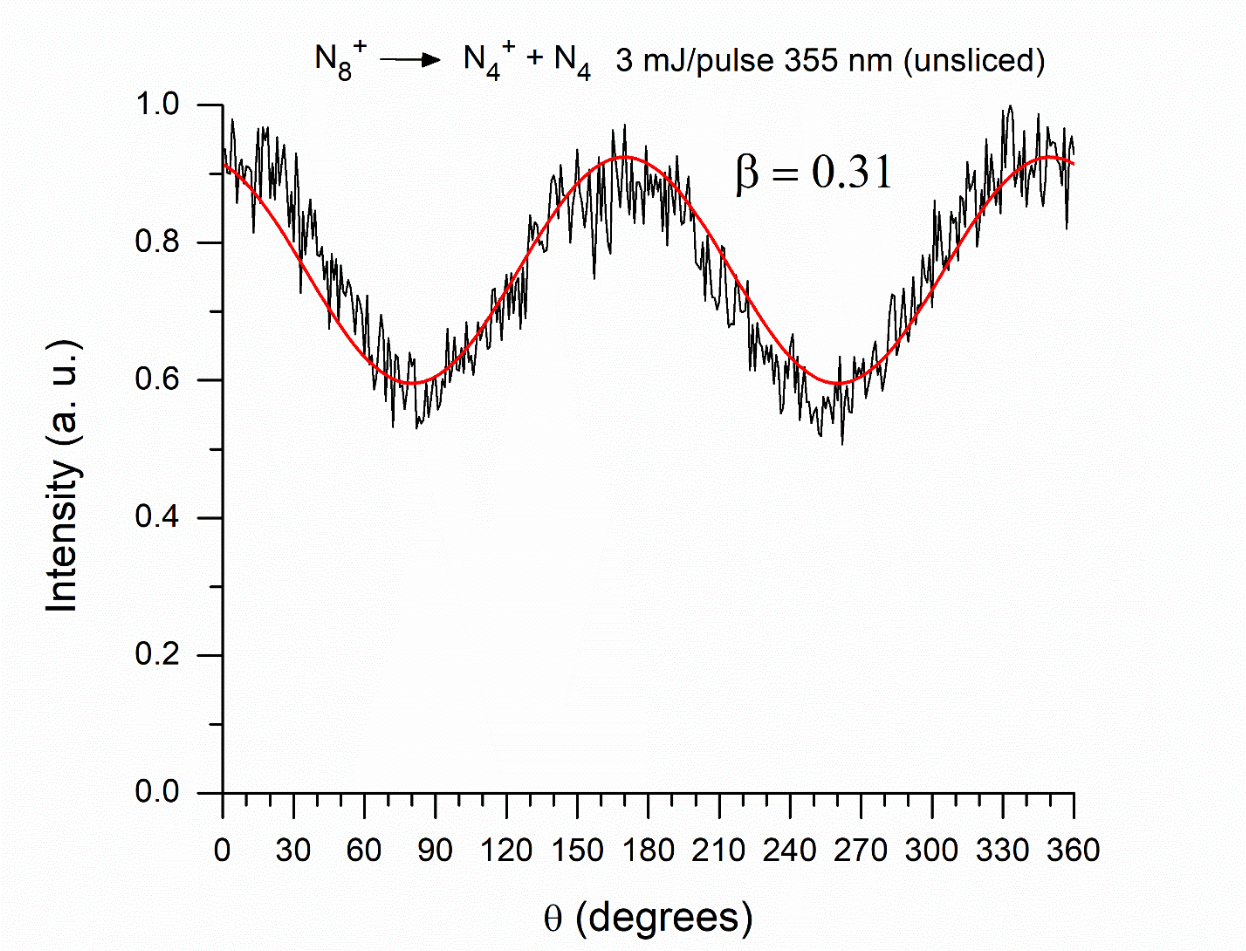

Figure S7. The angular distribution of the unsliced N_4^+ photofragment image from the dissociation of N_8^+ with vertically polarized light at 355 nm. The red line is a fit with ß = 0.31.

$A = 8.86218 \pm 0.03855$

$B = 0.31125 \pm 0.00809$

$C = -0.17440 \pm 0.01192$

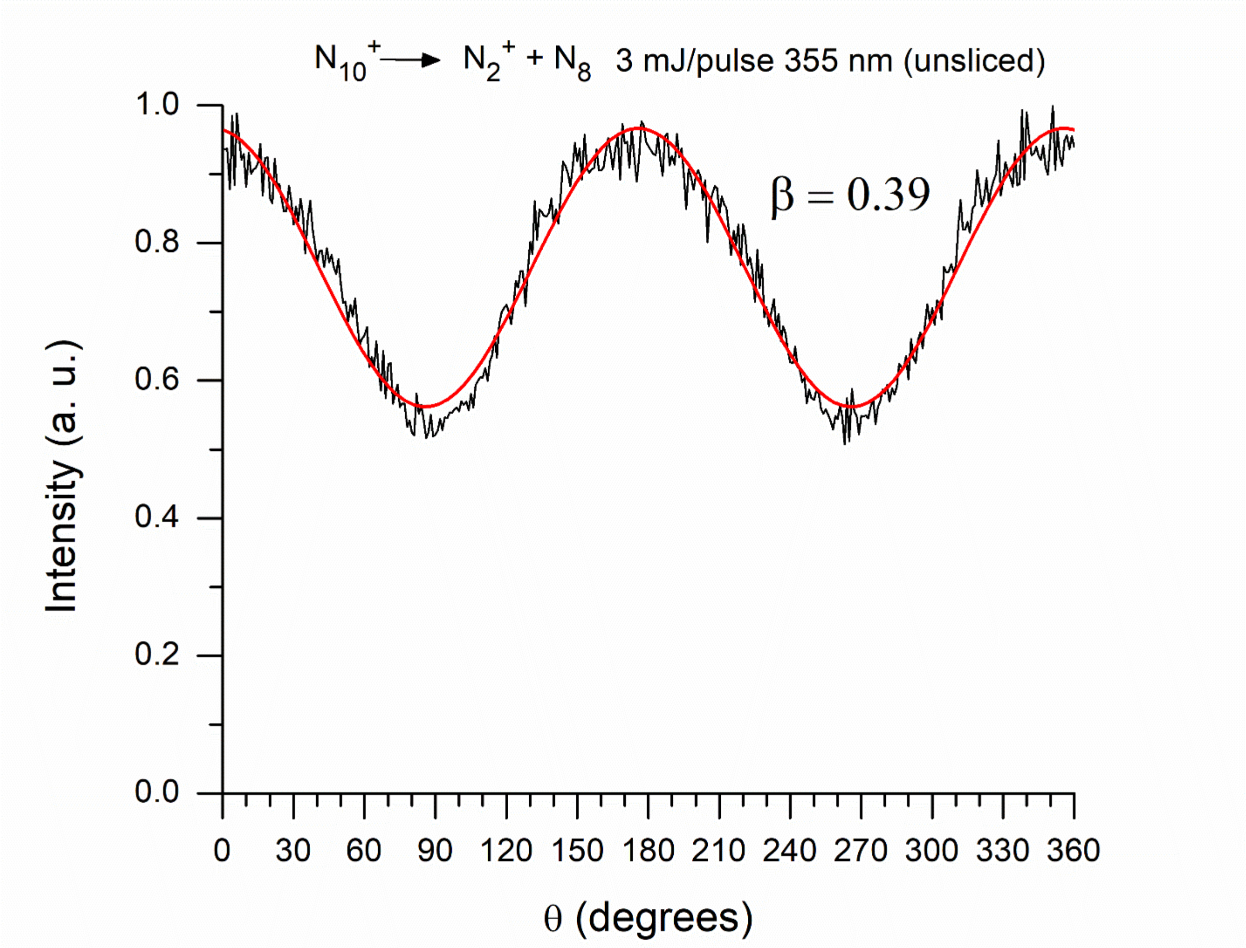

Figure S8. The angular distribution of the unsliced N_2^+ photofragment image from the dissociation of N_{10}^+ with vertically polarized light at 355 nm. The red line is a fit with ß = 0.39.

A = 8.75721 ± 0.02249

B = 0.38742 ± 0.00489

C = − 0.07364 ± 0.00565

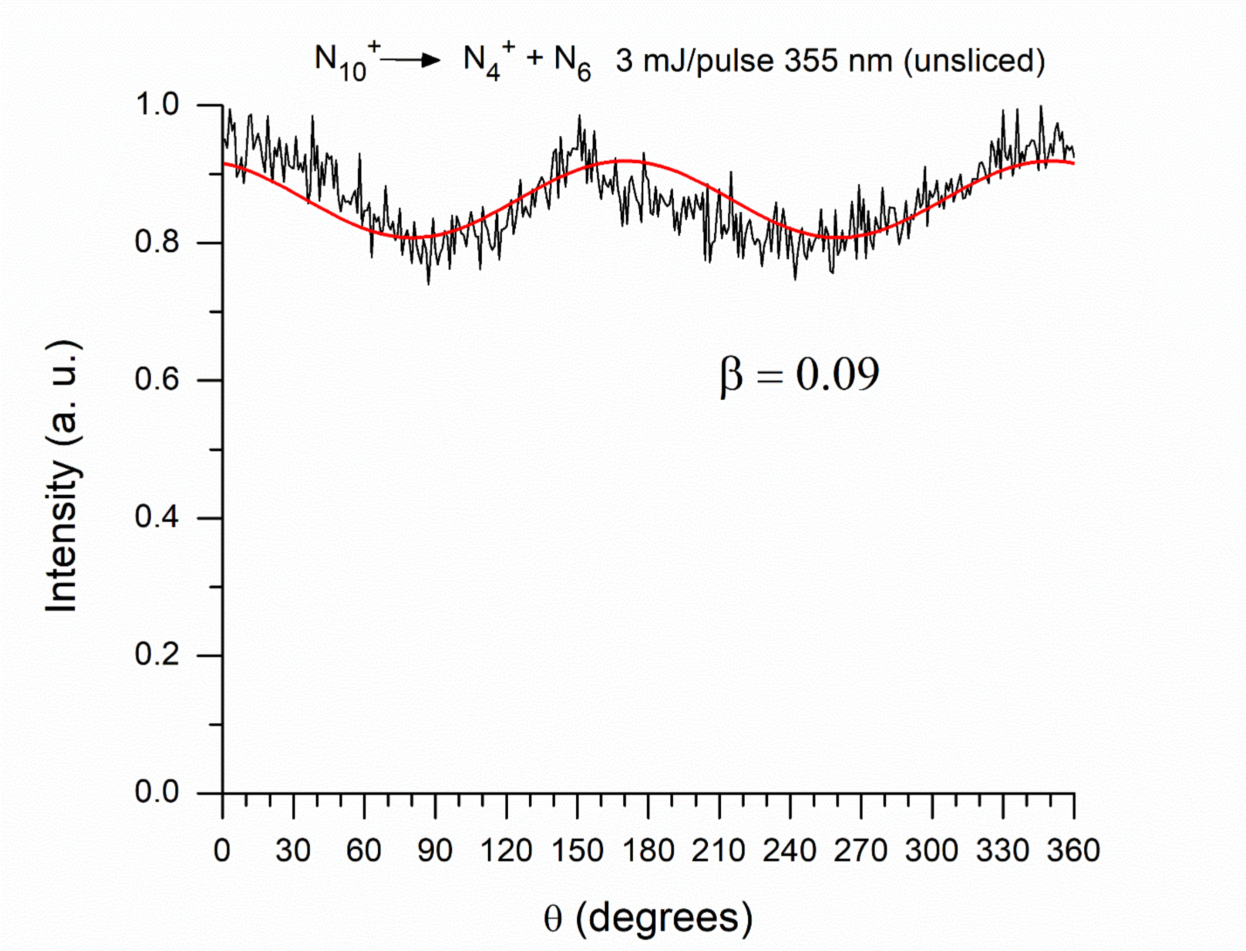

Figure S9. The angular distribution of the unsliced N_4^+ photofragment image from the dissociation of N_{10}^+ with vertically polarized light at 355 nm. The red line is a fit with ß = 0.09.

$A = 10.61818 \pm 0.02915$

$B = 0.08818 \pm 0.00479$

$C = -0.17334 \pm 0.02655$

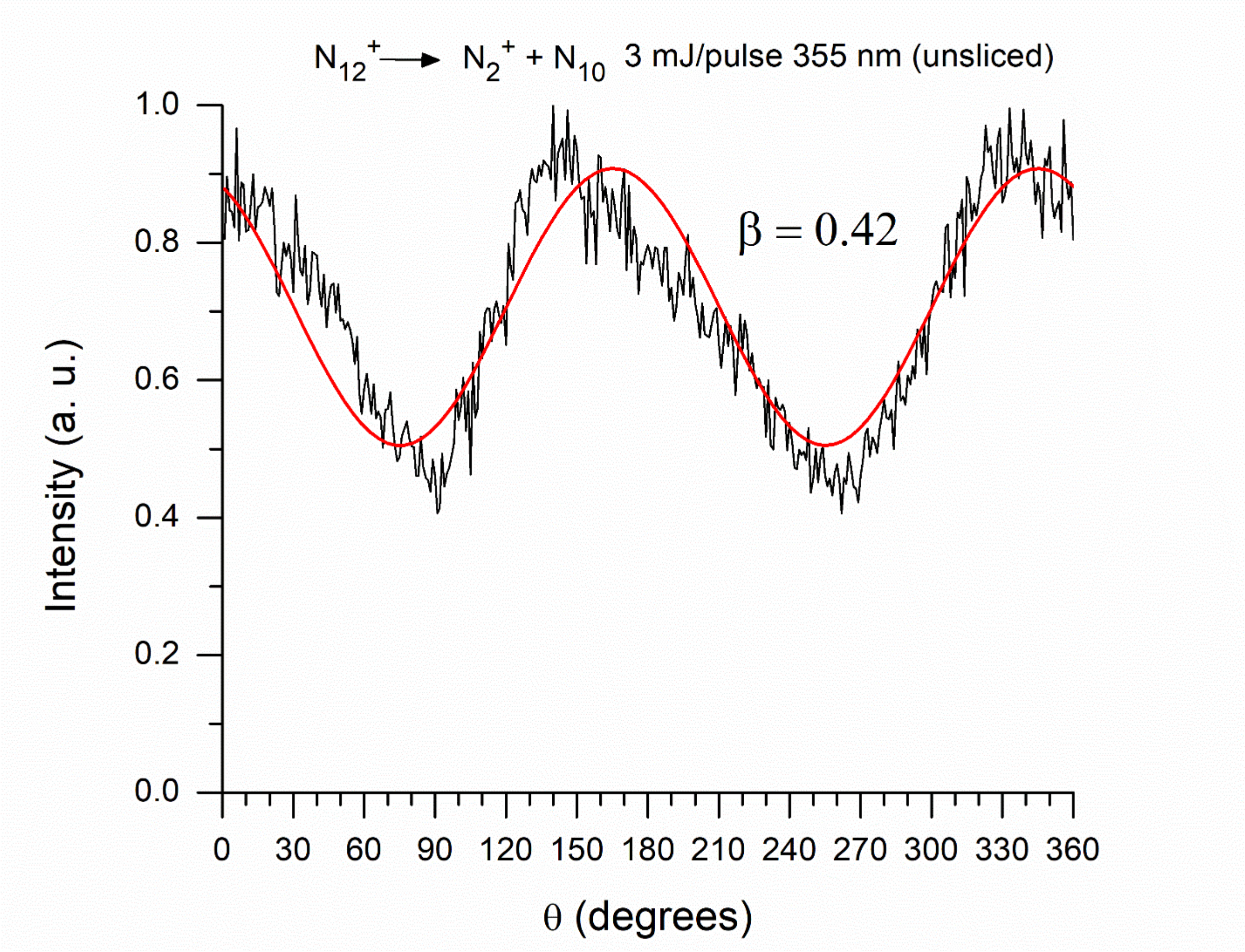

Figure S10. The angular distribution of the unsliced N_2^+ photofragment image from the dissociation of N_{12}^+ with vertically polarized light at 355 nm. The red line is a fit with ß = 0.42.

A = 8.03461 $\pm$ 0.04974

B = 0.4205 $\pm$ 0.01190

C = $-$0.25819 $\pm$ 0.01256

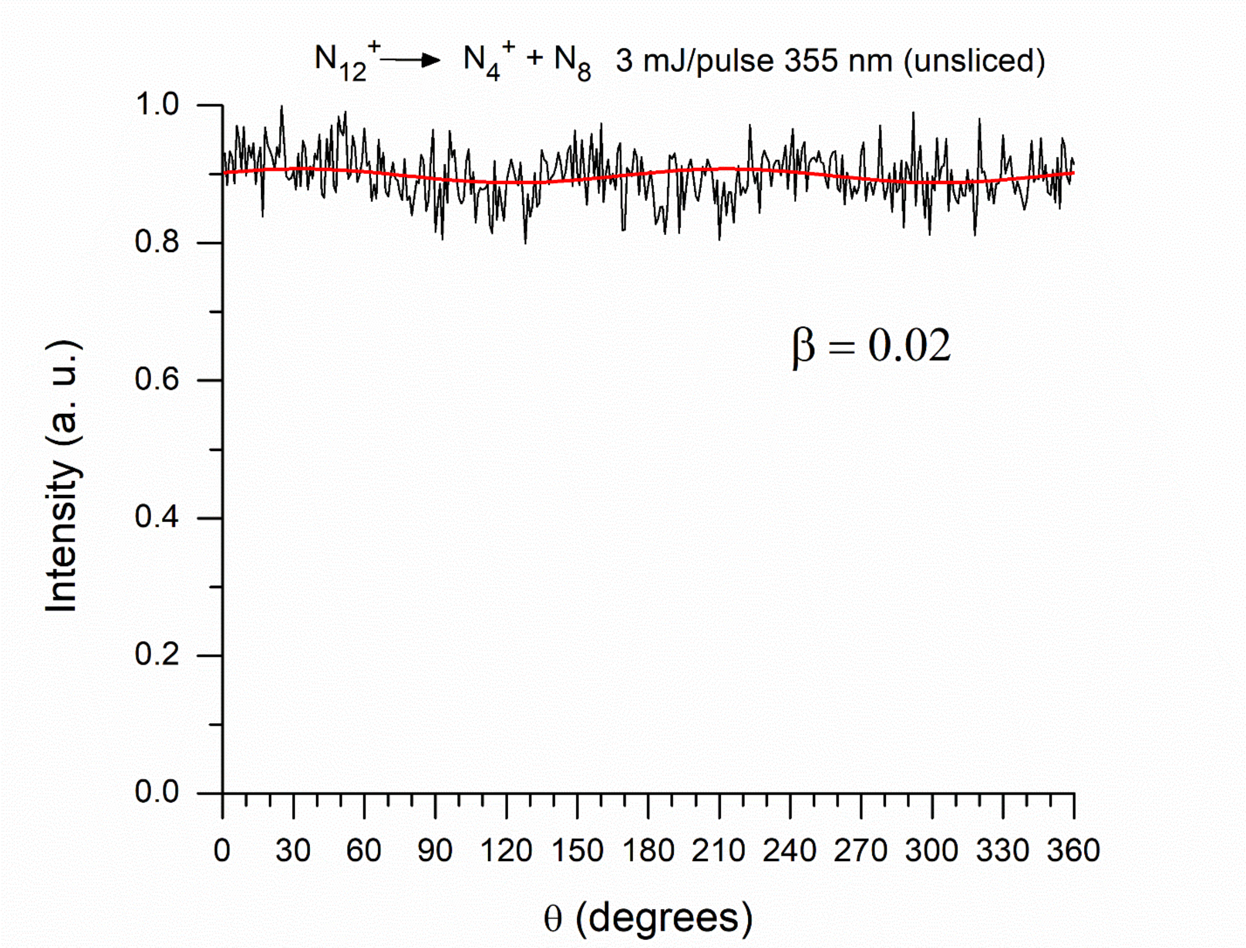

Figure S11. The angular distribution of the unsliced N_4^{+} photofragment image from the dissociation of N_{12}^{+} with vertically polarized light at 355 nm. The red line is a fit with ß = 0.02.

A = 11.23917 $\pm$ 0.02652

B = 0.01512 $\pm$ 0.00404

C = 0.57416 $\pm$ 0.13313

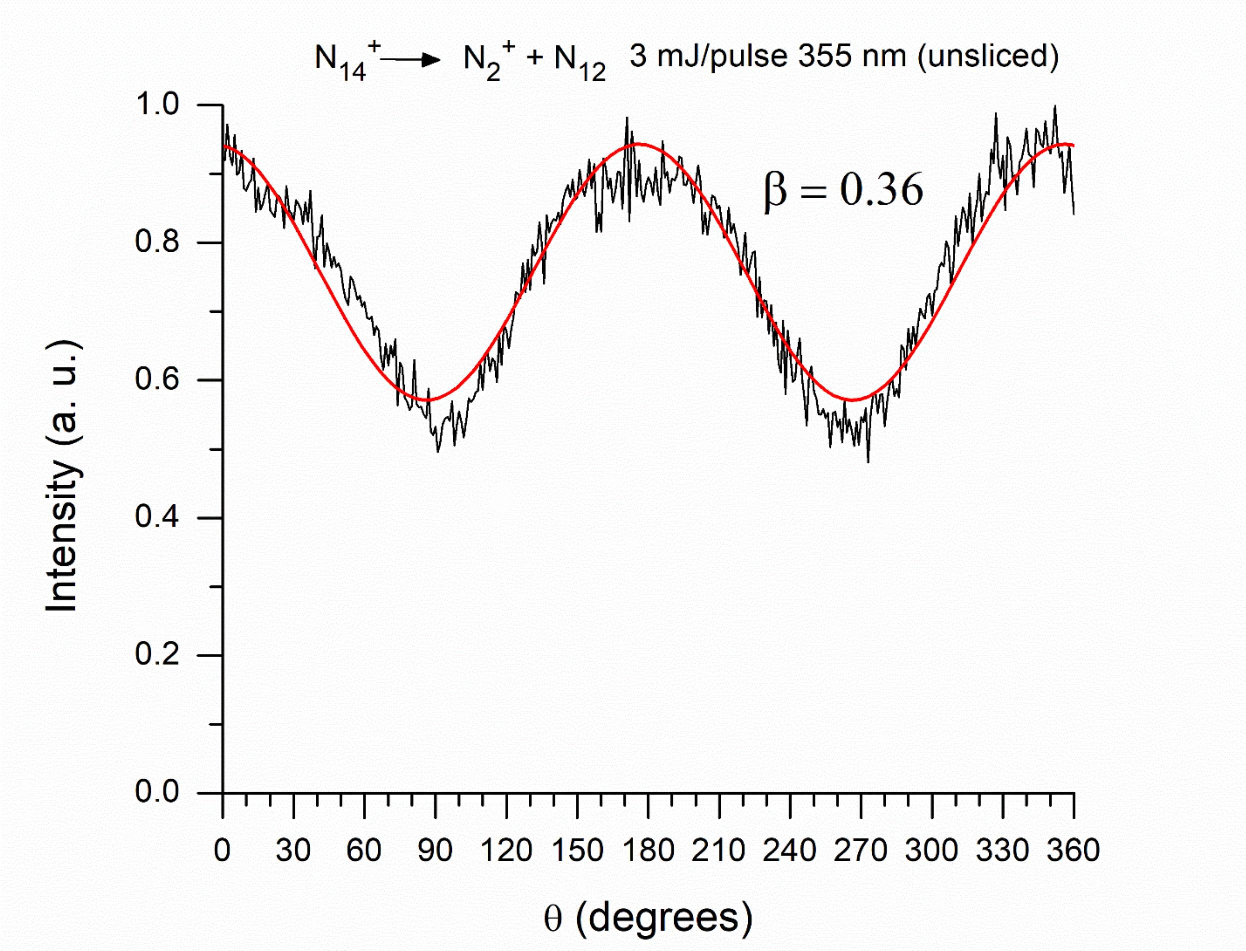

Figure S12. The angular distribution of the unsliced N_2^+ photofragment image from the dissociation of N_{14}^+ with vertically polarized light at 355 nm. The red line is a fit with ß = 0.36.

$A = 8.73681 \pm 0.03080$

$B = 0.35695 \pm 0.00665$

$C = -0.06639 \pm 0.00842$

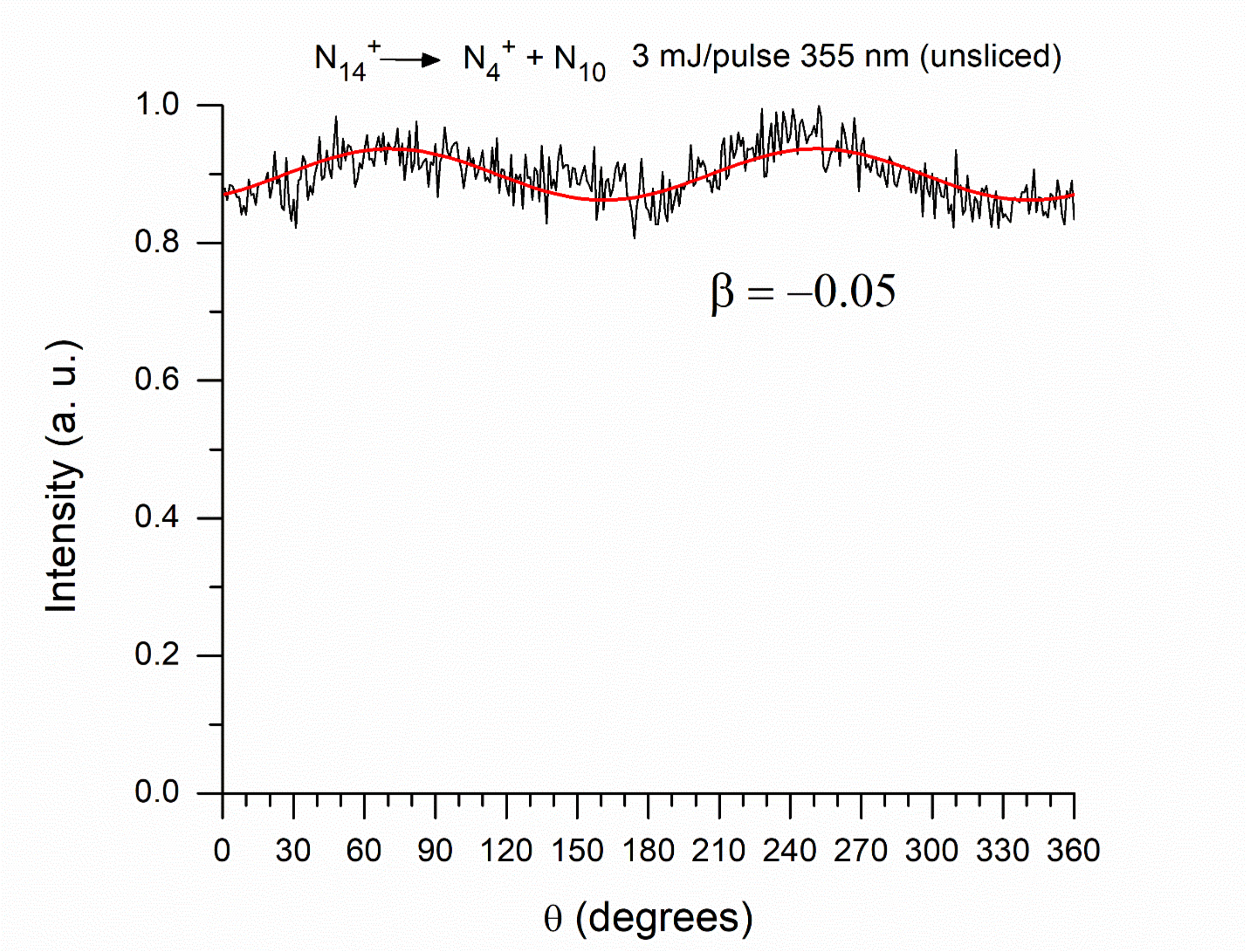

Figure S13. The angular distribution of the unsliced N_4^+ photofragment image from the dissociation of N_{14}^+ with vertically polarized light at 355 nm. The red line is a fit with ß = −0.05.

A = 11.46266 ± 0.02063

B = −0.05451 ± 0.00303

C = −0.32827 ± 0.02815

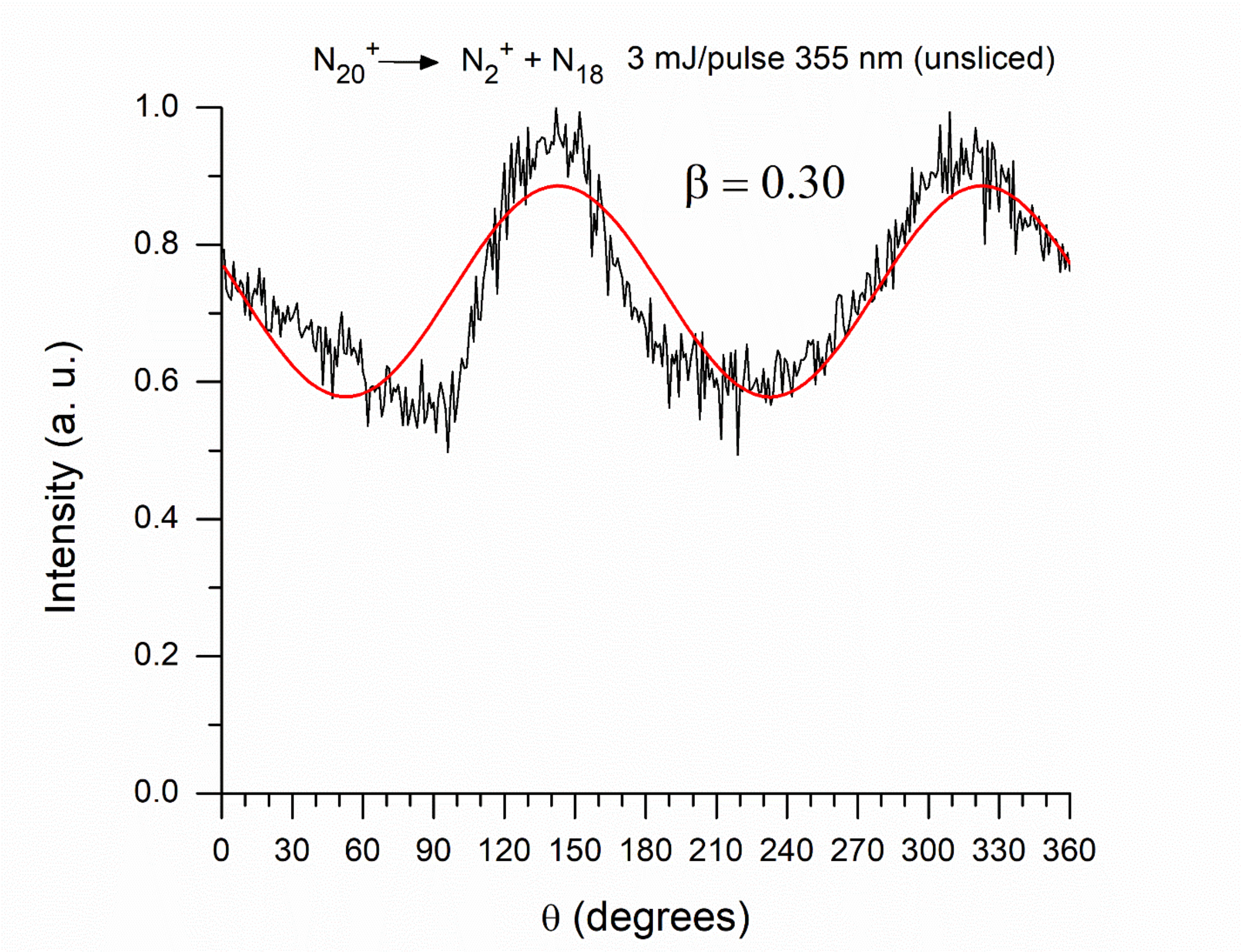

Figure S14. The angular distribution of the unsliced N_2^+ photofragment image from the dissociation of N_{20}^+ with vertically polarized light at 355 nm. The red line is a fit with ß = 0.30.

A = 8.55423 ± 0.04753

B = 0.30175 ± 0.01030

C = − 0.65026 ± 0.01570

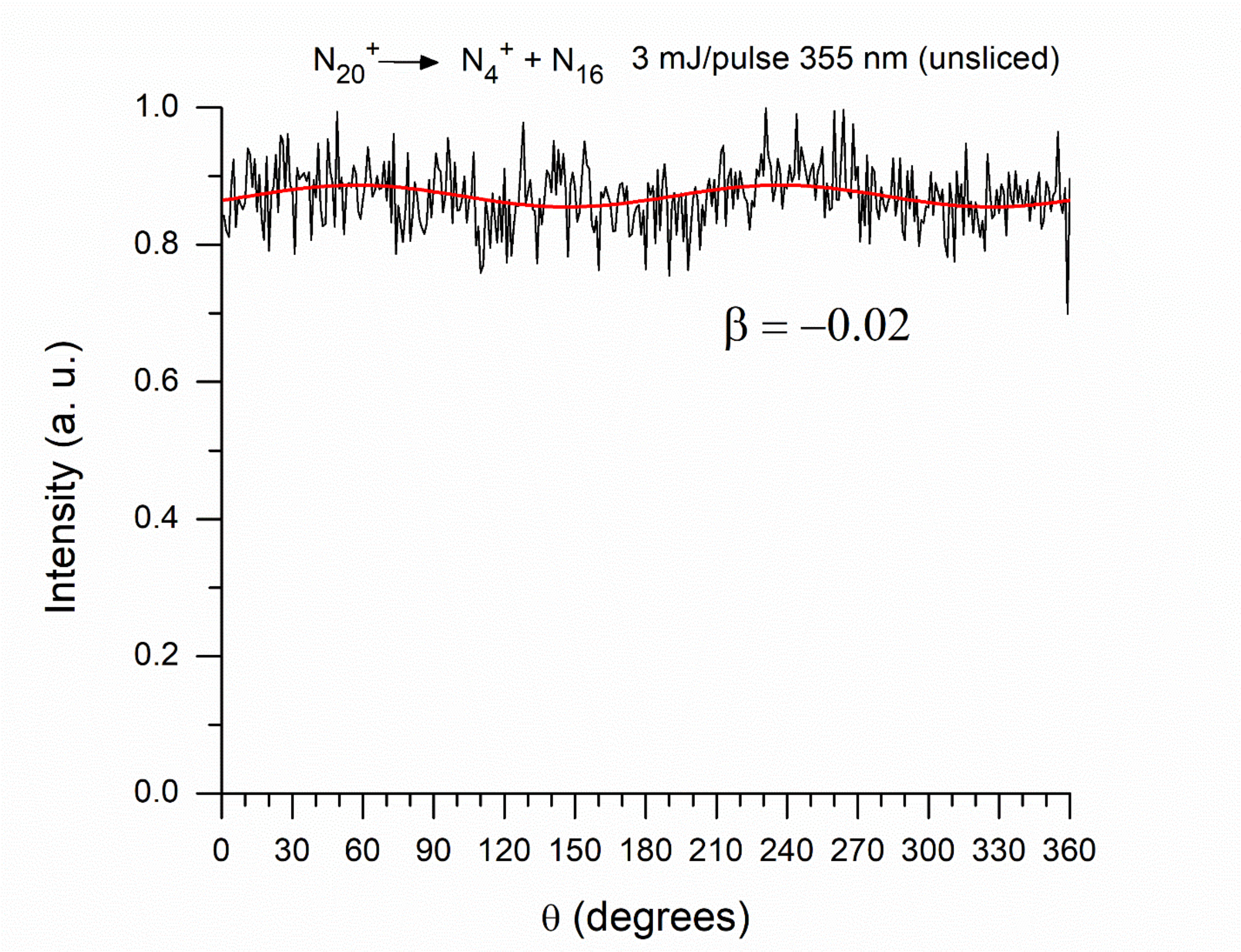

Figure S15. The angular distribution of the unsliced N_4^+ photofragment image from the dissociation of N_{20}^+ with vertically polarized light at 355 nm. The red line is a fit with ß = 0.02.

A = 11.01325 $\pm$ 0.03265

B = $-$0.02418 $\pm$ 0.00503

C = $-$0.57707 $\pm$ 0.10456

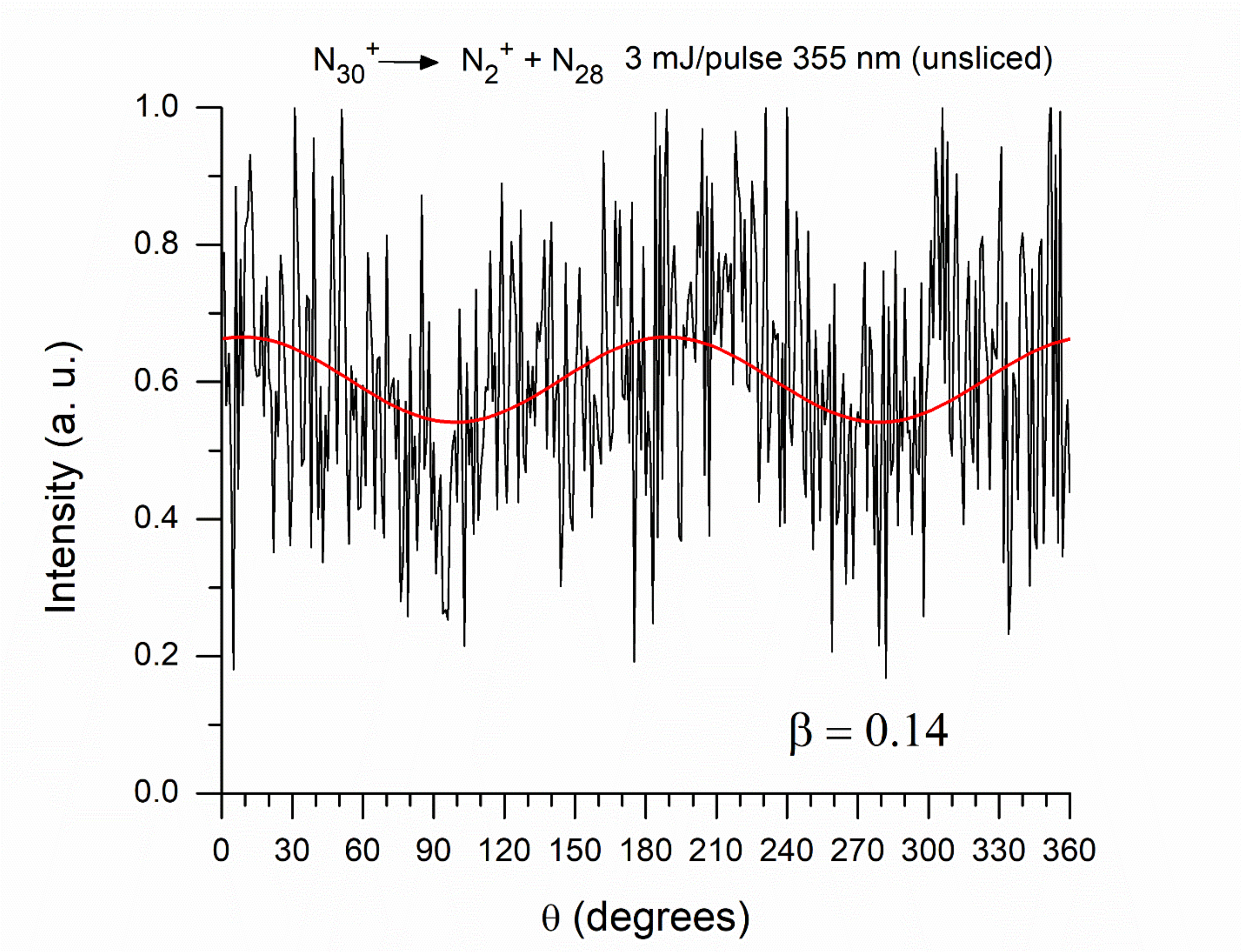

Figure S16. The angular distribution of the unsliced N_2^+ photofragment image from the dissociation of N_{30}^+ with vertically polarized light at 355 nm. The red line is a fit with ß = 0.14.

A = 7.32077 $\pm$ 0.13090

B = 0.14241 $\pm$ 0.03167

C = 0.15930 $\pm$ 0.10708

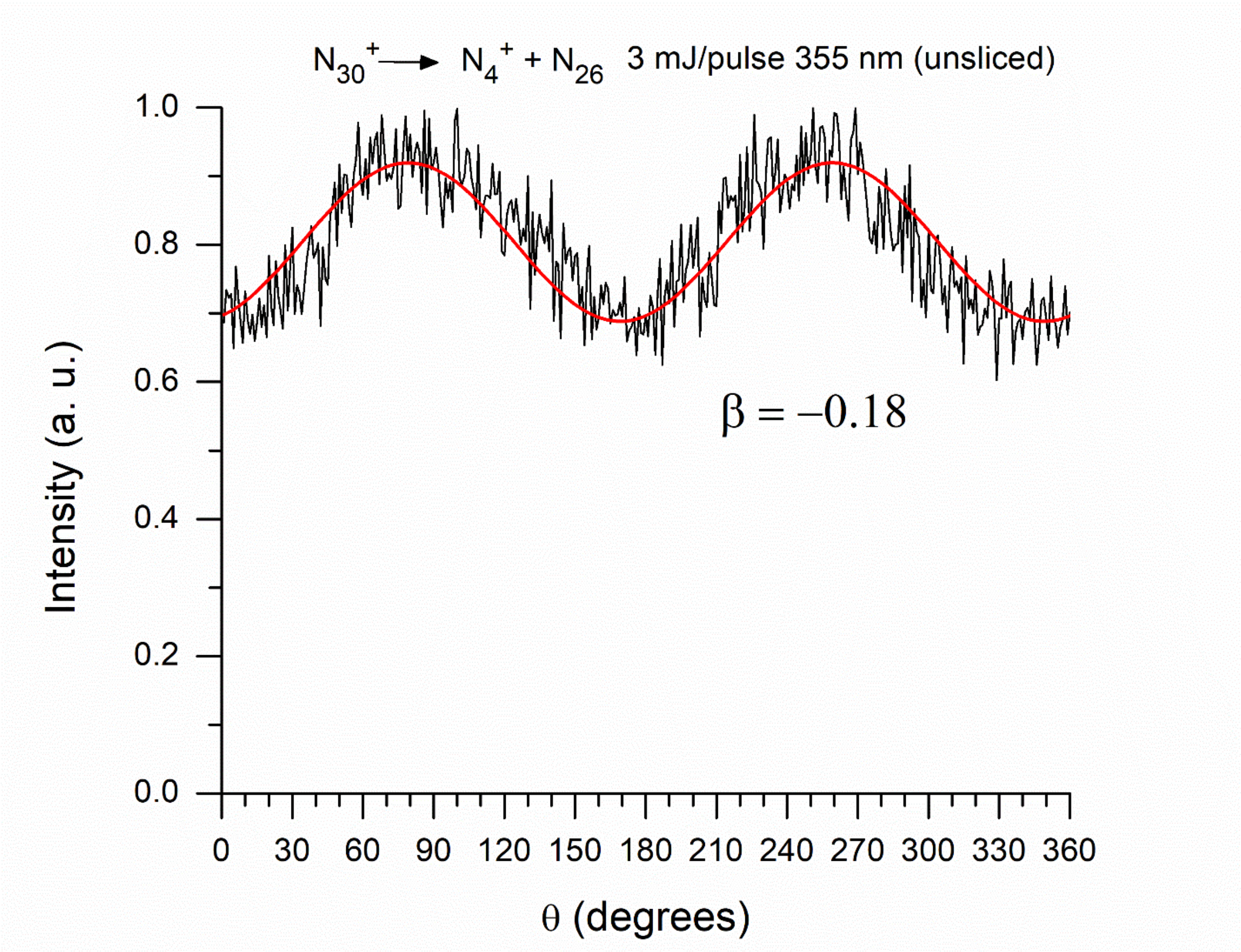

Figure S17. The angular distribution of the unsliced N_4^+ photofragment image from the dissociation of N_{30}^+ with vertically polarized light at 355 nm. The red line is a fit with ß = − 0.18.

A = 10.58720 ± 0.03870

B = − 0.18305 ± 0.00598

C = − 0.18950 ± 0.01703

Kinetic Energy Determination for Multi-Nitrogen Cluster Dissociation

Given the size of the clusters imaged in this experiment, it is crucial to examine the validity of our usual kinetic energy analysis which models the photodissociation event as an explosion with two fragments. In this simple case, the law of conservation of linear momentum necessitates that both the charged fragment that is detected, and the uncharged co-fragment must have equal but opposite momenta in the plane of the detector. Using the time between photodissociation and detection, and the radial distance of the detected fragment from the center of the image, the velocity of the fragment (in the plane of the detector) is determined. Using conservation of momentum, the following relation is obtained:

$$v_c = -\frac{m_f v_f}{m_c} \tag{1}$$

where m_f and v_f correspond to the mass and velocity of the detected fragment, and m_c, v_c are the corresponding properties of the undetected co-fragment. Using eqn. 1, algebra and the definition of kinetic energy, the total kinetic energy of both fragments can be written as:

$$KE = \frac{1}{2} m_f {v_f}^2 \left(1 + \frac{m_f}{m_c}\right) \tag{2}$$

which is used to relate every point of the image to a kinetic energy, and through integration over angle at each radius yields a kinetic energy release (KER) spectrum.

In the present case, there is no reason to believe that the undetected mass of these nitrogen clusters remains intact as one distinct neutral fragment. However, the aforementioned analysis method remains useful, with additional limitations. Observe the definition of the center of mass of an N-particle system relative to the same origin:

$$\overrightarrow{CM} = \frac{1}{M} \sum_{i=1}^{N} m_i \vec{r_i} \tag{3}$$

where M is the sum of all individual particle masses m_i and $\vec{r_i}$ is the position of mass m_i with respect to an arbitrary reference origin.[1] By definition of momentum and the properties of derivatives, the sum of the momenta of each of these particles can be written as:

$$\vec{P} = \sum_{i=1}^{N} \vec{p_i} = \sum_{i=1}^{N} \frac{d(m_i \vec{r_i})}{dt} = \frac{d}{dt} \sum_{i=1}^{N} m_i \vec{r_i} = M \frac{d(\overrightarrow{CM})}{dt} = M\overrightarrow{V_{CM}} \tag{4}$$

with $\overrightarrow{V_{CM}}$ representing the time derivative of the center of mass, equivalent to the velocity of the center of mass.[1] Then, in the model of photodissociation, there exists a detected fragment and a system of co-fragments for which a center of mass can be determined. Using the component versions of the above equations, since the instrument can only measure displacement in the plane of the detector, a familiar equation appears from momentum conservation:

$$v_{CM} = -\frac{m_f v_f}{M_c} \tag{5}$$

where v_{CM} is the velocity of the center of mass of all co-fragments excluding the detected fragment and M_c is the sum of all co-fragment masses; this equation is analogous to eqn. 1 where only one co-fragment exists. The difference between these scenarios rears its head when determining the total kinetic energy of the system. The total kinetic energy of an N-particle system is given by:

$$KE = \frac{1}{2}\sum_{i=1}^{N} m_i {v_i}^2 = \frac{1}{2}\sum_{i=1}^{N} m_i(\overrightarrow{v_i} \cdot \overrightarrow{v_i}) \tag{6}$$

where v_i is the velocity of the particle with mass m_i to an arbitrary reference origin.[1,2] From the principle of superposition, it is possible to deconstruct the velocity of each particle into its velocity with respect to the center of mass ($\overrightarrow{v_{i,cm}}$) and the center of mass velocity with respect to the reference origin ($\overrightarrow{V_{CM}}$).[2] Carrying out the vector dot product multiplication produces:

$$KE = \frac{1}{2}\sum_{i=1}^{N} m_i\left[\left(\overrightarrow{v_{i,cm}} + \overrightarrow{V_{CM}}\right) \cdot \left(\overrightarrow{v_{i,cm}} + \overrightarrow{V_{CM}}\right)\right] \tag{7}$$

$$= \frac{1}{2}\sum_{i=1}^{N} m_i\left(\overrightarrow{v_{i,cm}} \cdot \overrightarrow{v_{i,cm}}\right) + \frac{1}{2}\sum_{i=1}^{N} 2m_i\left(\overrightarrow{v_{i,cm}} \cdot \overrightarrow{V_{CM}}\right) + \frac{1}{2}\sum_{i=1}^{N} m_i\left(\overrightarrow{V_{CM}} \cdot \overrightarrow{V_{CM}}\right) \tag{8}$$

$$= \frac{1}{2}\sum_{i=1}^{N} m_i {v_i}^2 + \left(\sum_{i=1}^{N} m_i \overrightarrow{v_{i,cm}}\right) \cdot \overrightarrow{V_{CM}} + \frac{1}{2}\sum_{i=1}^{N} m_i {V_{CM}}^2 \tag{9}$$

and from this the middle term disappears as the first vector in that dot product can be shown to be the zero vector:[2]

$$
\begin{aligned}
\sum_{i=1}^{N} m_i \overrightarrow{v_{i,cm}} &= \sum_{i=1}^{N} m_i\left(\overrightarrow{v_i} - \overrightarrow{V_{CM}}\right) \\
&= \sum_{i=1}^{N} m_i \overrightarrow{v_i} - \left(\sum_{i=1}^{N} m_i\right) \overrightarrow{V_{CM}} \\
&= \sum_{i=1}^{N} m_i \frac{d\overrightarrow{r_i}}{dt} - M\overrightarrow{V_{CM}} \\
&= \frac{d}{dt}\sum_{i=1}^{N} m_i \overrightarrow{r_i} - M\overrightarrow{V_{CM}} \\
&= M\overrightarrow{V_{CM}} - M\overrightarrow{V_{CM}} \\
&= \vec{0} \qquad (10)
\end{aligned}
$$

by using the result from eqn. 4.[1,2] Hence:[2]

$$
\begin{aligned}
KE &= \frac{1}{2}\sum_{i=1}^{N} m_i v_i^{\,2} + \frac{1}{2}\sum_{i=1}^{N} m_i V_{CM}^{\;2} = \frac{1}{2}\sum_{i=1}^{N} m_i v_i^{\,2} + \frac{1}{2}\left(\sum_{i=1}^{N} m_i\right) V_{CM}^{\;2} \\
&= \frac{1}{2}\sum_{i=1}^{N} m_i v_i^{\,2} + \frac{1}{2} M V_{CM}^{\;2}. \qquad (11)
\end{aligned}
$$

Therefore, the total kinetic energy of an N-particle system is the sum of the kinetic energy provided by the motion of its center of mass and the kinetic energy from the motion of the individual particles with respect to the center of mass, which is observed as the system expanding or contracting while the center of mass travels through space.[2]

Returning to the photodissociation model, the total kinetic energy from motion in the plane of the detector is written as:

$$
\begin{aligned}
KE &= \frac{1}{2} m_f v_f^{\,2} + \frac{1}{2} M_c v_{CM}^{\;2} + \frac{1}{2}\sum_{i=1}^{N} m_{c,i} v_{c,i}^{\;2} \\
&= \frac{1}{2} m_f v_f^{\,2} + \frac{1}{2} M_c \left(-\frac{m_f v_f}{M_c}\right)^2 + \frac{1}{2}\sum_{i=1}^{N} m_{c,i} v_{c,i}^{\;2} \\
&= \frac{1}{2} m_f v_f^{\,2}\left(1 + \frac{m_f}{M_c}\right) + \frac{1}{2}\sum_{i=1}^{N} m_{c,i} v_{c,i}^{\;2} \qquad (12)
\end{aligned}
$$

where $v_{c,i}$ is the velocity of a co-fragment of mass $m_{c,i}$ in the plane of the detector, and eqn. 5 is used to substitute for v_{CM}. It's clear that the first term of this expression is analogous to the total kinetic energy of a two-fragment photodissociation system, but now there is a second term which

cannot be determined from the detection of a single fragment, and is thus unaccounted for in the kinetic energy release spectrum. Since this term is always greater than or equal to zero for all real physical phenomena, it must be true that the KER_{max} determined for any system where the co-fragment cannot safely be assumed as an intact rigid-body is an underestimation of the true value of KER_{max}.

References:

1. Taylor, J. R. "Momentum and Angular Momentum," in *Classical Mechanics*; Young, L.; University Science Books: Sausalito, 2005, pp. 83−104.
2. Taylor, J. R. "Rotational Motion of Rigid Bodies," in *Classical Mechanics*; Young, L.; University Science Books: Sausalito, 2005, pp. 367−416.

Computational Studies

Computations were carried out with the Gaussian16 program (this is the full citation for reference 69):

Gaussian 16, Revision C.01, Frisch, M. J.; Trucks, G. W.; Schlegel, H. B.; Scuseria, G. E.; Robb, M. A.; Cheeseman, J. R.; Scalmani, G.; Barone, V.; Petersson, G. A.; Nakatsuji, H.; Li, X.; Caricato, M.; Marenich, A. V.; Bloino, J.; Janesko, B. G.; Gomperts, R.; Mennucci, B.; Hratchian, H. P.; Ortiz, J. V.; Izmaylov, A. F.; Sonnenberg, J. L.; Williams-Young, D.; Ding, F.; Lipparini, F.; Egidi, F.; Goings, J.; Peng, B.; Petrone, A.; Henderson, T.; Ranasinghe, D.; Zakrzewski, V. G.; Gao, J.; Rega, N.; Zheng, G.; Liang, W.; Hada, M.; Ehara, M.; Toyota, K.; Fukuda, R.; Hasegawa, J.; Ishida, M.; Nakajima, T.; Honda, Y.; Kitao, O.; Nakai, H.; Vreven, T.; Throssell, K.; Montgomery, J. A., Jr.; Peralta, J. E.; Ogliaro, F.; Bearpark, M. J.; Heyd, J. J.; Brothers, E. N.; Kudin, K. N.; Staroverov, V. N.; Keith, T. A.; Kobayashi, R.; Normand, J.; Raghavachari, K.; Rendell, A. P.; Burant, J. C.; Iyengar, S. S.; Tomasi, J.; Cossi, M.; Millam, J. M.; Klene, M.; Adamo, C.; Cammi, R.; Ochterski, J. W.; Martin, R. L.; Morokuma, K.; Farkas, O.; Foresman, J. B.; Fox, D. J. Gaussian, Inc., Wallingford CT, 2016.

All calculations were carried out using MP2 or DFT/B3LYP with the aug-cc-pVTZ basis set. An “ultrafine” integration grid, and the optimization threshold for energy and structure optimizations were set to “tight.”

MP2 Computations

N_2^+
m = 2
Energy (Hartrees): -108.735547

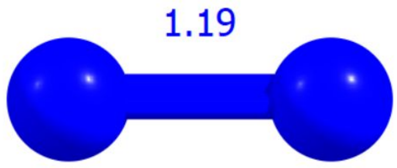

Coordinates:

7	0.000000000	0.000000000	0.594746000
7	0.000000000	0.000000000	-0.594746000

Frequencies (cm^{-1}):	Intensities:
1763.0760	0.0000

N_2
m = 1
Energy (Hartrees): -109.359818

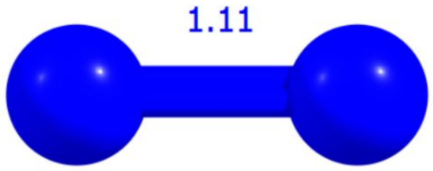

Coordinates:

7	0.000000000	0.000000000	0.557042000
7	0.000000000	0.000000000	-0.557042000

Frequencies (cm^{-1}):	Intensities:
2186.8364	0.0000

N_4^+ (Linear)
m = 2
Energy (Hartrees): -218.142956
$\langle S^2 \rangle = 0.7604$

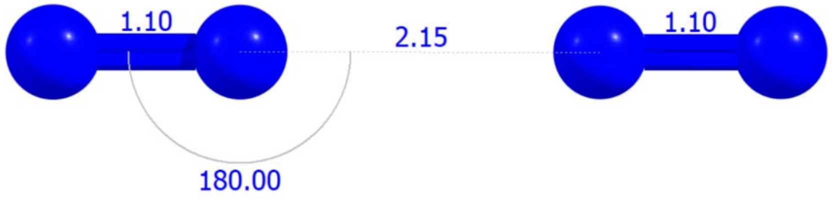

Coordinates:

7	0.000000000	0.000000000	-0.994481000
7	0.000000000	0.000000000	2.063085000
7	0.000000000	0.000000000	-2.063085000
7	0.000000000	0.000000000	0.994481000

Frequencies (cm^{-1}):	Intensities:
-260.5113	0.0000
-260.5113	0.0000
-120.7857	0.0027
-120.7857	0.0027
376.5801	0.0000
3481.1006	0.0000
3670.8599	0.0000

N_4^+ (Rhombus)
m = 2
Energy (Hartrees): -218.165756
$\langle S^2 \rangle = 0.7589$

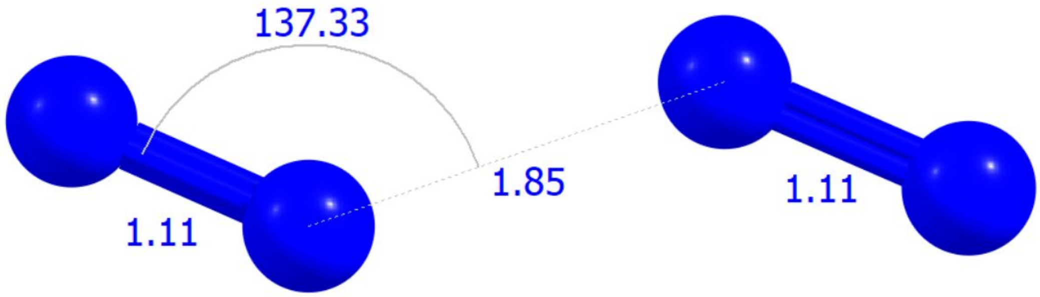

Coordinates:

7	1.888193000	-0.140285000	-0.000469000
7	-1.888197000	0.140276000	-0.000469000
7	0.875720000	0.302472000	0.000469000
7	-0.875716000	-0.302464000	0.000469000

Frequencies (cm^{-1}):	Intensities:
97.4640	0.7363
427.1432	0.0000
1457.5635	0.0049
1805.9275	8788.0963
2732.1150	0.0006
3169.9934	8558.5763

N_6^+ (Linear)
m = 2
Energy (Hartrees): -327.53751
$\langle S^2 \rangle = 0.7785$

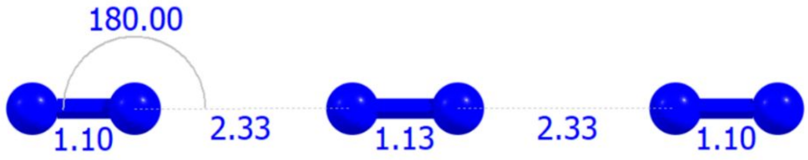

Coordinates:

7	0.000000000	0.000000000	2.896732000
7	0.000000000	0.000000000	-0.564893000
7	0.000000000	0.000000000	3.992664000
7	0.000000000	0.000000000	0.564896000
7	0.000000000	0.000000000	-3.992666000
7	0.000000000	0.000000000	-2.896733000

Frequencies (cm^{-1}):	Intensities:
-97.8557	0.0000
-97.8557	0.0000
-47.1261	0.1877
-47.1261	0.1877
108.0086	0.0000
108.0086	0.0000
186.8419	0.0000
330.7330	0.0000
330.7330	0.1877
1692.7333	0.1877
2108.2780	0.0000
3637.3176	0.0000
5346.9149	0.0000

N_6^+ (T)
m = 2
Energy (Hartrees): -327.531349
$\langle S^2 \rangle = 0.8602$

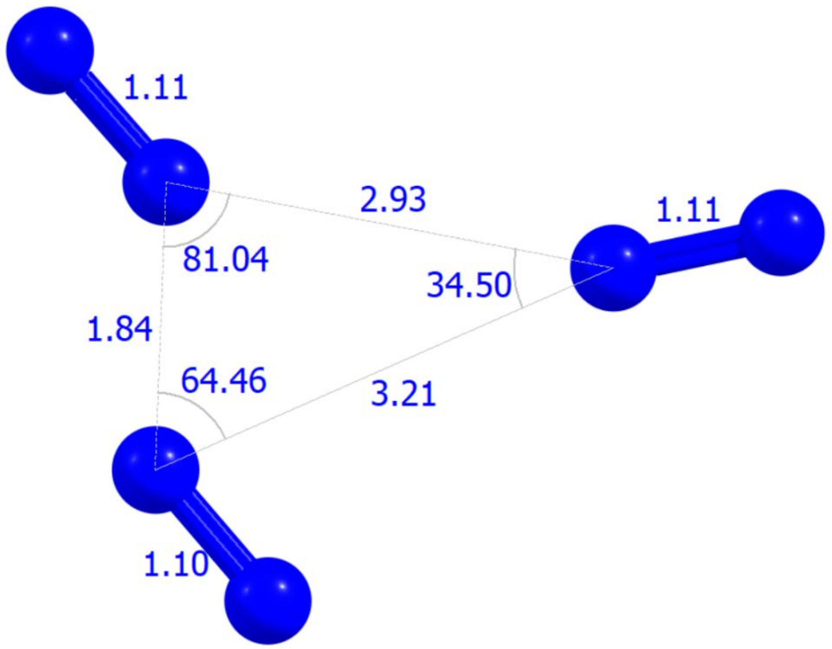

Coordinates:

7	-1.761869000	0.211173000	-0.000248000
7	1.120598000	0.760599000	0.017713000
7	-2.852230000	0.440086000	-0.002205000
7	1.849358000	1.591121000	-0.010002000
7	0.464683000	-1.922698000	0.002630000
7	1.179461000	-1.080281000	-0.007887000

Frequencies (cm^{-1}):	Intensities:	Frequencies (cm^{-1}):	Intensities:
19.4004	0.1781	1883.7666	12719.5642
45.9594	1.4583	2193.4603	1.2791
87.1330	0.4739	2785.6486	133.9709
92.0767	1.0741	3204.9701	15151.0123
93.3113	7.5624		
100.0932	0.6539		
438.3388	0.1935		
1491.4426	71.9399		

Table 1. Energetics computed for nitrogen cluster cations at the MP2/aug-cc-pVTZ level. Bond dissociation energies (BDE) were evaluated for N_4^+ and N_6^+ isomers using $N_4^+ \rightarrow N_2^+ + N_2$ and $N_6^+ \rightarrow N_4^+$(Linear) + N_2.

Molecule	Energy (Hartrees)	Rel. Energy (kcal/mol)	BDE (kcal/mol)
N_2	-109.359818		
N_2^+	-108.735547		
N_4^+(Linear)	-218.142956	+14.31	29.9
N_4^+(Rhombus)	-218.165756	0.00	44.2
N_6^+(Linear)	-327.53751	0.00	21.8
N_6^+(T)	-327.531349	+3.87	17.9

Note: The N_6^+ cluster obtained here has the charge distributed equally over the six nitrogens in the cluster, rather than an $N_4^+(N_2)$ configuration. We attempted to freeze the N_4^+ structure and add an N_2 to this, but these calculations did not converge.

Density Functional Theory Computations

N_2^+
m = 2
Energy (Hartrees): -108.983242

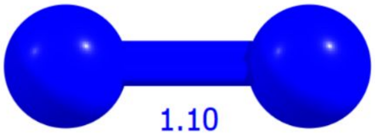

Coordinates:

7	0.000000000	0.000000000	0.552275000
7	0.000000000	0.000000000	-0.552275000

Frequencies (cm^{-1}):	Intensities:
2329.1267	0.0000

N_2
m = 1
Energy (Hartrees): -109.565010

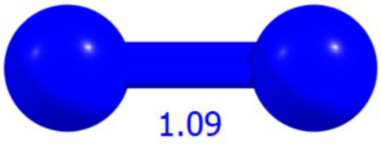

Coordinates:

7	0.000000000	0.000000000	0.545581000
7	0.000000000	0.000000000	-0.545581000

Frequencies (cm^{-1}):	Intensities:
2447.3286	0.0000

N_4^+
m = 2
Energy (Hartrees): -218.612432
$\langle S^2 \rangle$ = 0.7604

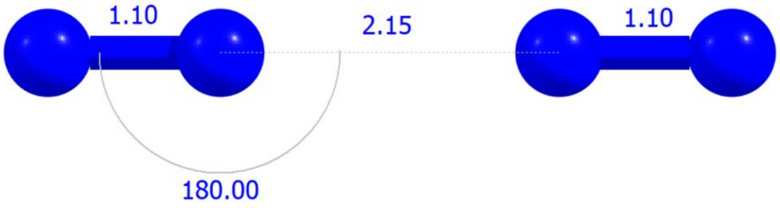

Coordinates:

7	0.000000000	0.000000000	1.077369000
7	0.000000000	0.000000000	-2.173639000
7	0.000000000	0.000000000	2.173639000
7	0.000000000	0.000000000	-1.077369000

Frequencies (cm^{-1}):	Intensities:
80.8237	0.0933
80.8237	0.0933
115.0169	0.0000
115.0169	0.0000
239.1131	0.0000
2387.9806	201.8255
2410.2561	0.0000

N_6^+ (Linear)
m = 2
Energy (Hartrees): -328.197236
$\langle S^2 \rangle = 0.7529$

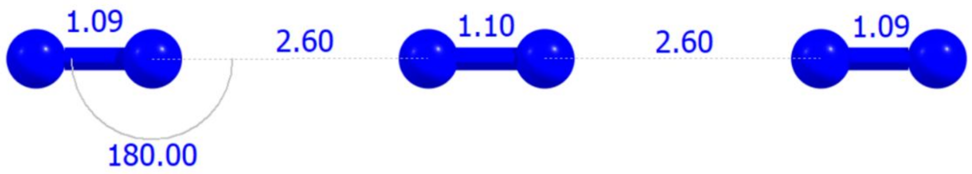

Coordinates:

7	0.000000000	0.000000000	3.148715000
7	0.000000000	0.000000000	-0.547905000
7	0.000000000	0.000000000	4.243080000
7	0.000000000	0.000000000	0.547905000
7	0.000000000	0.000000000	-4.243080000
7	0.000000000	0.000000000	-3.148715000

Frequencies (cm^{-1}):	Intensities:
14.5513	0.0233
14.5513	0.0233
72.7470	0.0000
72.7470	0.0000
75.5522	0.0264
75.5522	0.0264
98.9441	0.0000
126.3643	79.6764
133.4627	0.0000
133.4627	0.0000
2398.7414	0.0000
2412.0193	542.8371
2419.2121	0.0000

N_6^+ (Y)
m = 2
Energy (Hartrees): -328.192943
$\langle S^2 \rangle = 0.7582$

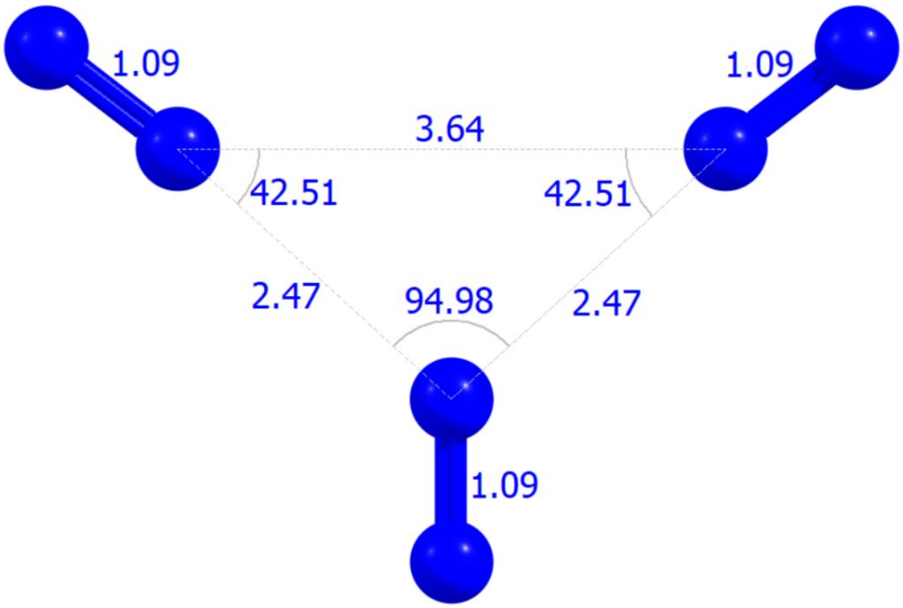

Coordinates:

7	1.819876000	0.515667000	-0.000017000
7	0.000009000	-1.152515000	-0.000002000
7	2.685912000	1.184199000	0.000026000
7	-0.000038000	-2.247254000	0.000046000
7	-2.685865000	1.184260000	0.000159000
7	-1.819895000	0.515643000	-0.000212000

Frequencies (cm^{-1}):	Intensities:	Frequencies (cm^{-1}):	Intensities:
39.1990	0.3066	152.8941	85.8769
80.8016	3.4933	2406.4495	93.3631
83.0881	0.0000	2413.2893	296.7508
94.0023	0.0001	2422.3031	7.2344
99.6292	23.5675		
107.3250	0.5373		
134.1607	0.1152		
151.9700	0.0166		

N_8^+ (Near D_{4h})
m = 2
Energy (Hartrees): -437.767389
$\langle S^2 \rangle$ = 0.7534

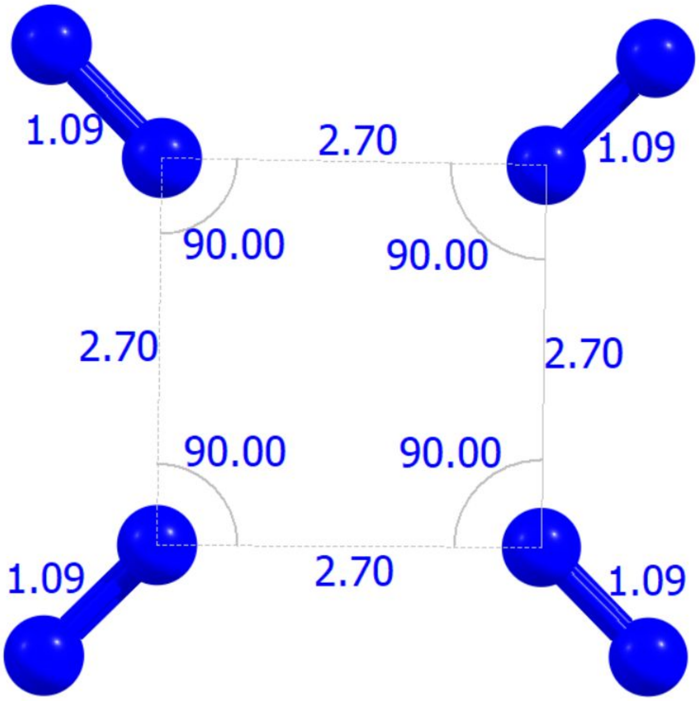

Coordinates:

7	-0.000017000	-1.910991000	0.000000000
7	1.910991000	-0.000045000	0.000000000
7	-0.000015000	-3.004488000	0.000000000
7	3.004488000	0.000056000	0.000000000
7	-3.004488000	0.000010000	0.000000000
7	-1.910991000	-0.000018000	0.000000000
7	0.000000000	1.910990000	0.000000000
7	0.000033000	3.004487000	0.000000000

Frequencies (cm^{-1}):	Intensities:
-1.1335	0.0000
59.7239	8.6960
59.7266	8.6914
60.3909	0.0003
65.5356	0.0000
71.5819	0.0001
82.0625	32.6687
82.0659	32.6763

85.5050	0.0000
85.5061	0.0000
95.6874	0.0000
97.9763	0.0000
106.6792	0.0000
141.3403	0.0000
2419.3453	228.9108
2419.3454	228.9108
2421.8014	0.0000
2426.2710	0.0000

N_8^+ (Linear)
m = 2
Energy (Hartrees): -437.774633
⟨S^2⟩ = 0.7526

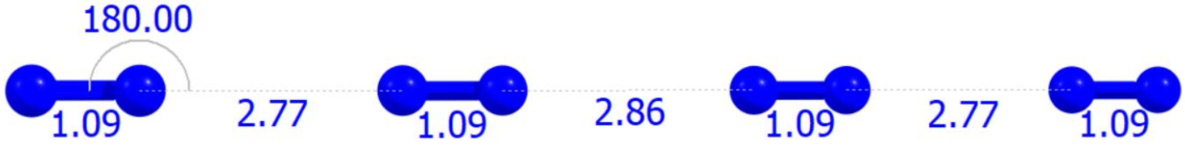

Coordinates:

7	0.000000000	0.000000000	5.294248000
7	0.000000000	0.000000000	1.431952000
7	0.000000000	0.000000000	6.387830000
7	0.000000000	0.000000000	2.525804000
7	0.000000000	0.000000000	-2.525804000
7	0.000000000	0.000000000	-1.431952000
7	0.000000000	0.000000000	-5.294248000
7	0.000000000	0.000000000	-6.387830000

Frequencies (cm^{-1}):	Intensities:	Frequencies (cm^{-1}):	Intensities:
9.3520	0.0021	2417.3780	0.0000
9.3520	0.0021	2421.0993	790.0077
18.0289	0.0000	2425.9352	0.0000
18.0289	0.0000		
53.0020	0.0000		
54.4132	0.0000		
54.4132	0.0000		
54.5997	0.0046		
54.5997	0.0046		
84.4145	0.0062		
84.4145	0.0062		
92.8633	66.9359		
93.9429	0.0000		
93.9429	0.0000		
116.4711	0.0000		
2416.6766	113.2760		

N_{10}^{+} (Near D_{2h})
m = 2
Energy (Hartrees): -547.341366
$\langle S^2 \rangle$ = 0.7536

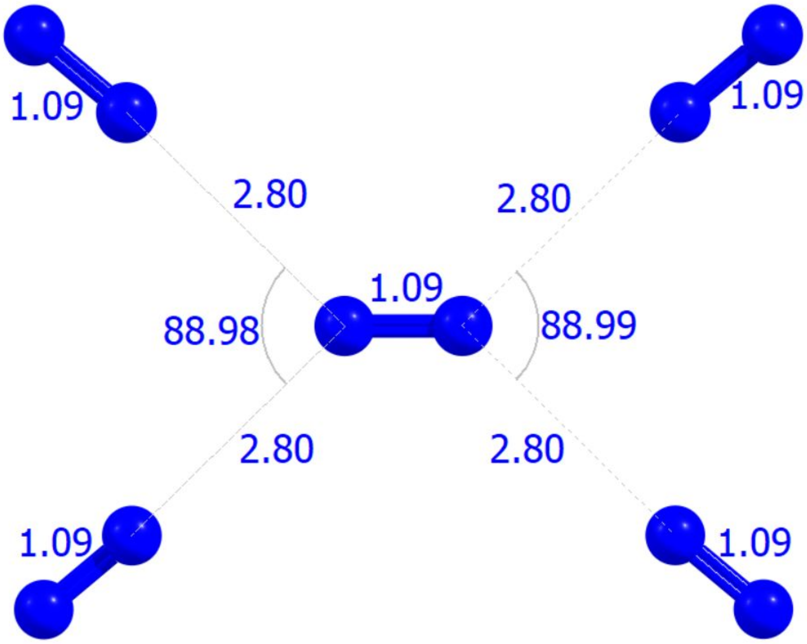

Coordinates:

7	-1.965482000	2.547602000	0.000000000
7	0.000000000	-0.547223000	0.000000000
7	-2.667769000	3.384696000	0.000000000
7	-0.000060000	0.547299000	0.000000000
7	2.667736000	-3.384585000	0.000000000
7	1.965416000	-2.547518000	0.000000000
7	-1.965097000	-2.547868000	0.000000000
7	-2.667291000	-3.385040000	0.000000000
7	1.965169000	2.547739000	0.000000000
7	2.667379000	3.384898000	0.000000000

Frequencies (cm^{-1}):	Intensities:
-3.9108	0.0000
12.6144	0.8168
19.4737	0.1577
20.0898	0.0000
22.8291	0.4738
56.7031	0.0010
56.9690	2.9663
60.2820	0.0000

63.8264	0.0000
64.7696	0.0000
65.4434	0.0000
65.4804	0.0000
68.0964	0.0887
74.0357	5.0486
77.4977	0.0000
98.5089	35.9771
103.2914	28.1515
124.4758	0.0000
127.1879	0.0000
2411.6895	0.0000
2428.7085	306.0945
2429.1611	0.0227
2429.5107	395.0290
2433.7258	0.0001

N_{10}^{+} (Linear)
m = 2
Energy (Hartrees): -547.348459
$\langle S^2 \rangle$ = 0.7521

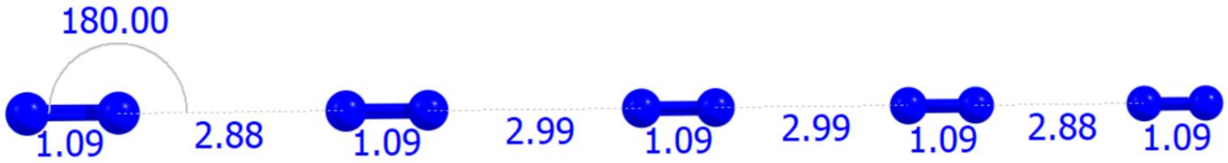

Coordinates:

7	0.000000000	0.000000000	3.525044000
7	0.000000000	0.000000000	-0.546439000
7	0.000000000	0.000000000	4.618297000
7	0.000000000	0.000000000	0.546439000
7	0.000000000	0.000000000	-4.618297000
7	0.000000000	0.000000000	-3.525044000
7	0.000000000	0.000000000	-7.484602000
7	0.000000000	0.000000000	-8.577701000
7	0.000000000	0.000000000	7.484602000
7	0.000000000	0.000000000	8.577701000

Frequencies (cm^{-1}):	Intensities:	Frequencies (cm^{-1}):	Intensities:
5.7590	0.0155	66.4562	0.0014
5.7590	0.0155	66.4562	0.0014
11.0166	0.0000	67.7441	0.0000
11.0166	0.0000	67.7441	0.0000
17.3610	0.0106	92.3828	0.0000
17.3610	0.0106	102.1092	40.6849
35.9126	0.0000	2422.5526	83.0179
42.5571	0.0000	2423.7464	0.0000
42.5571	0.0000	2425.9093	0.0000
42.7078	0.0017	2426.1247	1269.1363
42.7078	0.0017	2430.7532	0.0000
64.9450	37.4310		
65.7108	0.0000		
65.7108	0.0000		

Table 2. Energetics computed for nitrogen cluster cations at the B3LYP/aug-cc-pVTZ level. Dissociation energy was evaluated for N_4^+ and N_6^+ isomers using $N_4^+ \rightarrow N_2^+ + N_2$ and $N_6^+ \rightarrow N_4^+ + N_2$. The bond dissociation energies (BDE) are calculated for elimination of nitrogen to produce the next-smaller linear ions.

Molecule	Energy (Hartrees)	Relative Energy (kcal/mol)	BDE (kcal/mol)
N_2	-109.565010		
N_2^+	-108.983242		
N_4^+ (Linear)	-218.612432	0.00	40.3
N_6^+ (Linear)	-328.197236	0.00	12.4
N_6^+ (Y)	-328.192943	+2.69	9.73
N_8^+ (Linear)	-437.774633	0.000	7.77
N_8^+ (Near D_{4h})	-437.767389	+4.55	3.23
N_{10}^+ (Linear)	-547.348459	0.00	5.53
N_{10}^+ (Near D_{2h})	-547.341366	+4.45	1.08
